# Unraveling Spatiotemporal Transient Dynamics at the Nanoscale via Wavelet Transform-Based Kelvin Probe Force Microscopy

**DOI:** 10.1021/acsnano.3c06488

**Published:** 2023-10-25

**Authors:** Pardis Biglarbeigi, Alessio Morelli, Serene Pauly, Zidong Yu, Wenjun Jiang, Surbhi Sharma, Dewar Finlay, Amit Kumar, Navneet Soin, Amir Farokh Payam

**Affiliations:** † Nanotechnology and Integrated Bio-Engineering Centre (NIBEC), School of Engineering, Ulster University, York Street, Belfast BT15 1AP, Co. Antrim, Northern Ireland, United Kingdom; ⧧ School of Science and Engineering, University of Dundee, Nethergate, Dundee, DD1 4NH, Scotland, United Kingdom; § School of Mathematics and Physics, Queen’s University Belfast, University Road, Belfast BT7 1NN, Northern Ireland, United Kingdom; ∥ Institute for Materials Research and Innovation (IMRI), University of Bolton, Deane Road, Bolton BL3 5AB, United Kingdom; ⊥ College of Transportation Engineering, Dalian Maritime University, Dalian 116026, China; ζ Centre for New Energy Transition Research Technologies (CfNETR), Federation University Australia, Gippsland Campus, Churchill, Victoria 3810, Australia; # School of Science, Computing and Engineering Technologies, Swinburne University of Technology, P.O. Box 218, Hawthorn Victoria 3122, Australia

**Keywords:** Kelvin probe force microscopy (KPFM), time-resolved KPFM (tr-KPFM), wavelet transforms, surface photovoltage, transient quantification

## Abstract

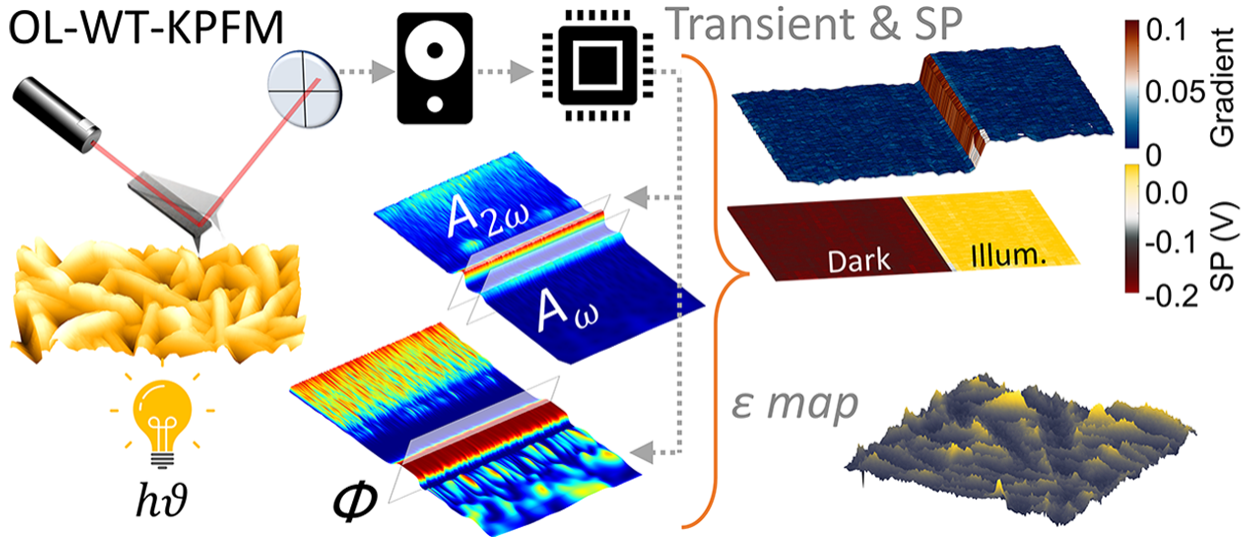

Mechanistic probing of surface potential changes arising from dynamic charge transport is the key to understanding and engineering increasingly complex nanoscale materials and devices. Spatiotemporal averaging in conventional heterodyne detection-based Kelvin probe force microscopy (KPFM) inherently limits its time resolution, causing an irretrievable loss of transient response and higher-order harmonics. Addressing this, we report a wavelet transform (WT)-based methodology capable of quantifying the sub-ms charge dynamics and probing the elusive transient response. The feedback-free, open-loop wavelet transform KPFM (OL-WT-KPFM) technique harnesses the WT’s ability to simultaneously extract spatial and temporal information from the photodetector signal to provide a dynamic mapping of surface potential, capacitance gradient, and dielectric constant at a temporal resolution 3 orders of magnitude higher than the lock-in time constant. We further demonstrate the method’s applicability to explore the surface-photovoltage-induced sub-ms hole-diffusion transient in bismuth oxyiodide semiconductor. The OL-WT-KPFM concept is readily applicable to commercial systems and can provide the underlying basis for the real-time analysis of transient electronic and electrochemical properties.

The emergence of cutting-edge technologies ranging from single-atom catalysis,^1^ strain-tuned electronics,^2^ photocapacitive/photofaradic bioelectronics,^3^ energy harvesting,^4^ and high-efficiency perovskite photovoltaics^5^ to synergistic human–machine interfaces^6^ is contingent on the development and understanding of advanced materials, including quantum, plasmonic, van der Waals two-dimensional heterostructures, and photonic materials.^7−11^ Both the static and dynamic nanoscale properties for such materials, including the surface potential (SP) distribution and its evolution, govern their interfacial interactions and subsequent physico-electrochemical properties. The spatiotemporal SP variations arising from the fundamental micro/nano-structural differences in work function, layer thickness/orientation,^12^ applied stimuli of charge insertion/removal,^13,14^ and charge generation/recombination have been captured in pristine materials as well in *in-operando* devices.^15−22^ These measurements have been enabled by advances in quantitative electrical scanning probe microscopy techniques, namely, scanning tunneling microscopy (STM), electrostatic force microscopy (EFM), and Kelvin probe force microscopy (KPFM). Considering the objective of correlating local electric potentials to topographical features, EFM is at best a semiquantitative tool; on the other hand, the interpretation of STM data is nontrivial, especially when the potential profile is unknown, posing the issue of differentiating the wave functions driven by the material’s behavior and by potential profile contours.^23,24^ Consequently, KPFM has become the *de facto* measurement technique, providing a quantitative measurement of the SP from contact potential difference (CPD) with high spatial resolution.

Particularly, the efforts to enhance photovoltaic and photocatalytic efficiency necessitate a comprehensive understanding of the nanoscale spatiotemporal dynamics of the intricate “energy pump and delivery” mechanism of the charge separation and transfer process.^25−27^ Although advanced spectroscopic tools like transient absorption, reflectance, and time-resolved X-ray spectroscopy have emerged to decipher these complexities, their demanding equipment requirements limit their accessibility.^25,28−30^ Furthermore, as these techniques primarily focus on temporal aspects, spatial understanding is often limited. Bulk time-resolved surface photovoltage (tr-SPV) measurements, while informative, average out critical events within the heterogeneous particles and nanoscale interfaces.^27,31,32^ This spatial comprehension of the charge separation and transfer mechanism is further compounded by the pronounced anisotropy in aggregates. To tackle this, it is crucial to directly examine spatiotemporal events at the particle and interface level. Scanning probe microscopy techniques, offering direct electric potential imaging and charge process monitoring, hold immense promise.^25−27,31−33^ With their high sensitivity and nanoscale resolution, KPFM-based SPV measurements provide a potent tool for understanding charge separation dynamics.

In this context, the classical closed-loop KPFM (CL-KPFM), reliant on feedback-based nullification of long-range electrostatic forces, extracts the fundamental electronic properties via the simultaneous application of AC and DC signals between the sample and the tip. Since the outlining concepts of KPFM by Nonnenmacher et al.,^34^ the field has witnessed a significant increase in its sensitivity and resolution, leading to absolute quantification of charge densities and capacitance gradients,^35−37^ as well as the elimination of artifacts and inherent crosstalk.^37−39^ However, the CL-KPFM measurements limit the temporal resolution and lose higher-order harmonic information in favor of spatial resolution. These limitations arise from (i) the detection mechanisms of lock-in amplifier (LIA) or phase-locked loop (PLL), which requires multiple oscillations (typically ∼100s μs–5 ms) for demodulation, (ii) irretrievable loss of higher-order harmonics and transient responses, and (iii) the cantilever response time itself (typically the ms time scale), which combined with the feedback-loop time constant prohibits signal detection below this limit.^40,41^ While the common approach would be to seek high-speed LIAs, these too encounter limitations due to increased noise bandwidth and necessitate low-pass filtering to address phase mismatch, resulting in information loss, bandwidth limitation, and loss of transient response. Recent advancements propose alternative demodulation techniques including synchronous (LIA-based mixing),^42^ asynchronous (peak-hold),^43^ and amplitude/phase estimators using Lyapunov^44^ and Kalman^45^ filter approaches, aiming to enhance the measurement speed.^46^ However, mixing can introduce upper sideband components, if the filtering is inadequate, posing challenges to information integrity. Kalman and Lyapunov filters, although capable of compensating for this issue, exhibit substantial implementation complexity and are sensitive to noise beyond the carrier frequency. A comprehensive exploration of these high-speed demodulation techniques can be found in Ruppert et al.^46^ Nevertheless, these purported techniques primarily expedite amplitude estimation for linear systems and mechanisms but fall short in capturing dynamic information concerning cantilever–surface interactions. Notably, they are ill-suited for complex multidimensional measurements encompassing nonlinearities, multifrequency analysis, mode coupling, and transient response assessment. These aspects are, however, indispensable for characterizing material properties and demand innovative approaches for accessing transient information. Furthermore, beyond LIA-based hardware limitations, measurement errors stemming from parasitic influences, incorrect phase configurations, feedback gains, and dynamic electronic properties operating faster than KPFM measurement time scales can lead to considerable experimental discrepancies of around hundreds of millivolts from the actual CPD.^41^ Thus, for understanding the temporal electrodynamics of increasingly complex nanoscale materials, while the standard CL-KPFM fails completely, the choice of high-speed LIAs and demodulation techniques is equally fraught with implementation challenges.

Since the report on closed-loop, quantitative tr-SPV KPFM measurements by Sadewasser et al.,^47^ several approaches have been proposed in pursuit of high temporal resolution via the utilization of nonlinear electrostatic tip–sample interaction forces. For instance, through the integration of a pump–probe excitation to KPFM, ultrafast open- and closed-loop SP measurements have been performed,^48−51^ by applying short voltage pulses to the tip (modulated by a slower sinusoidal envelope) in synchronization with a square impulse waveform to the sample. In the closed-loop scheme, the complex pump–probe KPFM setup relies on single-frequency heterodyne detection and bias feedback and, hence, is subject to the standard assumptions required for conventional KPFM operation. Despite its highest claimed temporal resolution and ability to probe transients,^48,51^ the technique of using precise electrical impulses to probe samples is not universally applicable. For instance, photovoltaics and perovskites require light as the stimuli. Conversely, intensity-modulated KPFM and pulsed-illumination KPFM measurements exploit the nonlinearities of either cantilever frequency on photovoltage or photovoltage on the intensity of illumination despite the photocapacitance being linearly dependent on the illumination.^52−54^ Strelcov et al. reported an extension of the time-resolved KPFM (tr-KPFM), where the conventional dual-pass KPFM was used via the application of a high-frequency AC signal to the tip and a four-step low-frequency probing voltage waveform to the sample.^55^ This allowed the extraction of SP time dynamics at ON/OFF states at 10 ms temporal resolution.^55^ Based on the nullification concept, Collins et al. proposed a technique called electrochemical force microscopy (EcFM),^56^ wherein by applying a single-frequency excitation waveform to the tip and superimposing the positive, negative, and zero bias pulses, the excitation and relaxation processes are recorded during the ON/OFF states in a measurement window of 50 ms. Given the low temporal resolution, this technique, too, cannot be used to detect and quantify fast dynamics. Based on the integration of dual harmonic open-loop KPFM and the general acquisition mode (G-mode), Collins et al. have proposed a series of KPFM modalities including the general mode (G-mode) KPFM^41,57^ and the fast free force recovery (F3R) KPFM,^40^ respectively. The G-mode collects and compresses the photodetector signal at a sampling rate of 4 MHz, allowing the capture of cantilever–sample interaction dynamics with significant noise attenuation. While the reported temporal resolution of 66 μs is faster than the cantilever bandwidth, the accuracy of quantification may be reduced owing to the fitting-based approach for CPD determination. Additionally, the use of fast Fourier transform (FFT) to process the data leads to an averaged spectrum integrated over the whole acquisition time, invariably resulting in the loss of transient information. It should be noted that both the G-mode and F3R-KPFM techniques are inherently limited by the cantilever frequency, and owing to the use of FFT, their capability in the dynamics’ detection is limited by the period of the captured amplitude signals. Therefore, to analyze the complete dynamics of the nonstationary signals from the photodetector, an approach combining time- and frequency-domain analysis is necessary. The wavelet transform (WT) method overcomes these constraints by using the wavelet, which is defined as small oscillations with a quick decay, as the basis function.^58^ WT can analyze the dynamic response of the complicated nonlinear mechanisms by decomposing the signal into sets of dominant and subordinate features and owing to its time and frequency localization is an effective tool to analyze noisy and nonstationary signals.^59^

Here, we report the development of a time-resolved, open-loop wavelet-transform KPFM (OL-WT-KPFM) technique, capable of probing the time dynamics and SP transients in biased-OFF/ON states at a 1 μs temporal resolution with a high spatial resolution (as the standard amplitude-modulated KPFM). Through the proposed technique, all the transient and dynamic phenomena in the range of data acquisition board (DAQ) sampling frequencies, occurring at the sample surface, are captured. Our proposed computational approach brings together the dual harmonics open-loop KPFM and wavelet transform, allowing simultaneous nanoscale measurement of SP, capacitance gradient (*∂C/∂z*), and dielectric constant (ε) of materials without the need for multiple LIAs. Moreover, by employing principal component analysis (PCA) as an initial denoising methodology on the raw photodetector signal and owing to the inherent nature of the WT in providing time–frequency analysis, the time–frequency decomposition process can be applied directly to the signal without the loss of data experienced in the G-mode. To validate the technique, initial temporal resolution measurements were carried out on a Au pad of a KPFM calibration sample with a time-varying pulsed bias input. We further undertook dynamic probing of a low-bandgap, n*-*type semiconductor bismuth oxyiodide (BiOI) to capture surface photovoltage-induced sub*-*ms surface diffusion of charge carriers, consistent with the bulk time-resolved surface photovoltage measurements. By eliminating the closed-loop nullification, the associated LIA time constant, and the averaging errors associated with the often-used FFT-based implementations, we can fully capture the transient response and the higher harmonics of the cantilever motion. Consequently, all physical parameters including SP, capacitance gradient, and dielectric constant can be derived at a much higher temporal resolution, beyond the LIA time constant, thereby overcoming the limitations of nullifying, feedback-based system.

## Results and Discussion

**Figure 1:**
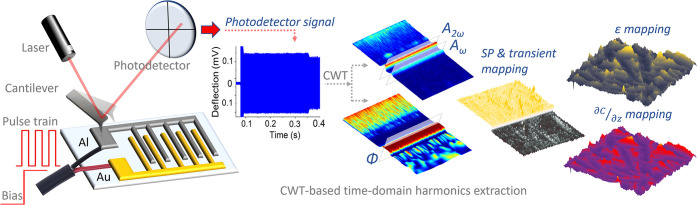
OL-WT-KPFM principle. Schematic of the WT-based KPFM measurements highlighting the key experimental and computational aspects: high-speed data acquisition of the photodetector signal, extraction of amplitude scalogram and phase map via CWT, selection of first (*A*_ω_) and second (*A*_2*ω*_) harmonics and phase (φ_ω_), calculation of the surface potential (SP) and mapping of capacitance gradient and dielectric constant, respectively. The WT method provides high temporal resolution (1 μs, currently limited by the DAQ's sampling frequency) of a feedback-free method to capture the transient and dynamic phenomenon at a spatial resolution of the standard AM-KPFM technique.

The proposed OL-WT-KPFM method relies on the retrieval of cantilever motion characteristics using continuous wavelet transform (CWT) analysis on the captured photodetector data stream (Figure 1). The OL-WT-KPFM implementation is similar to the conventional dual-pass KPFM, with two important distinctions: cantilever excitation at ω_AC_ during the lift mode (with *V*_DC_ = 0, i.e., no feedback) must be less than (where ω_0_ is the cantilever resonant frequency), and the resulting cantilever motion is to be acquired directly as a vertical deflection signal from the photodetector and captured via the DAQ. The choice of ω_AC_, the excitation frequency in lift mode, is dictated by the need to avoid the influence of cantilever dynamics and harmonic coupling that can affect the *V*_CPD_ quantification accuracy. Further details on the judicious choice of ω_AC_ is provided later in the results section and the Supporting Information. To acquire topography in the first pass, the cantilever is mechanically driven at its resonant frequency, ω_0_, while in the lift pass, an electrical AC drive, ω_AC_, of 3 V_p-p_ (1.5 V_AC_) at 15 kHz is utilized. In this lift pass, the cantilever vibrates freely in response to the periodic electrostatic forces arising from the interactions between the probe and the surface. The CWT-led analysis of the resultant photodetector data stream provides complete information about the amplitude and phase of the cantilever. It should be noted that the term “amplitude” here corresponds to the amplitude of the cantilever oscillations arising from the tip–sample interactions and the open-loop nature of the measurements, which are otherwise nulled by the Kelvin feedback controller in the CL-KPFM measurements.

Extracting the first- and second-harmonic responses (*A*_ω_, *A*_2ω_) and the phase (φ_ω_) at ω_AC_, the local *V*_CPD_ is calculated as
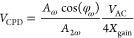
1where *X*_gain_ = *G*(ω)/*G*(2ω), *V*_AC_ is the applied AC drive signal, while φ_ω_ represents the phase between the cantilever response and the applied drive signal (see the Methods section).^37^

**Figure 2:**
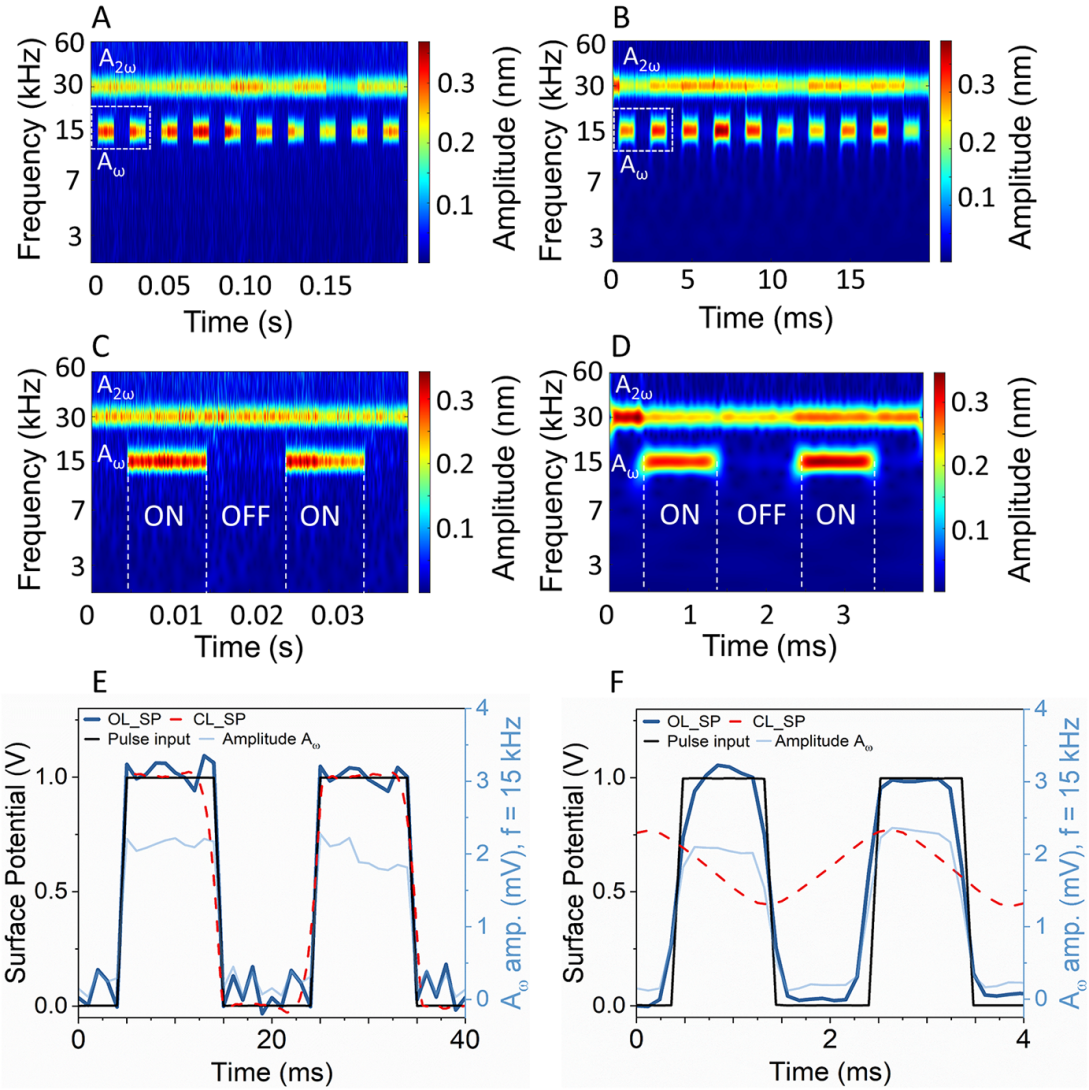
Comparison of CL-KPFM and OL-WT-KPFM temporal resolution. CWT-derived amplitude scalograms of the raw photodetector signal for the 50% duty cycle and 1 V_p-p_ pulse signal of (A, C) 20 ms and (B, D) 2 ms pulse periods, respectively. The scalograms show the temporal variation of the extracted *A*_ω_ (at 15 kHz applied AC signal) and the subsequently calculated open-loop surface potential (OL-SP). (E) For the 20 ms pulse period, while the OL-WT-KPFM was able to reconstruct the applied pulse, for the (F) 2 ms pulse, an obvious undersampling of results of the applied signal to a sinusoidal waveform occurs, while the OL-WT-KPFM was able to successfully reconstruct the applied pulses. The dotted boxes in (A, B) highlight the zoomed-in area shown in the images corresponding to (C, D) scalograms.

To ensure that the carrier dynamics do not influence the temporal resolution measurements, a featureless area on the Au pad of the KPFM calibration sample was chosen on which the CL-KPFM and OL-WT-KPFM measurements were sequentially carried out (see Figure S1). A pseudotemporal potential contrast was created using a pulse bias signal (1 V_p-p_, 0.1–2 kHz at 50% duty cycle, marked as a pulse train in Figure 1) applied to the Au pad. By applying the 1 V bias at 20 (Figure 2(A, C)) and 2 ms (Figure 2(B, D)) pulse periods, respectively, we first evaluated the ability of OL-WT-KPFM to reconstruct the applied pulse bias in comparison with the conventional CL-KPFM. It should be noted that both closed- and open-loop KPFM measurements are prone to noise, which can arise from factors including thermally induced cantilever noise, optical beam/cantilever deflection sensor noise, mechanical and environmental noise, electromagnetic/electrical interference, and finally, crosstalk between the channels.^60,61^ In our work, while the mechanical and environmental noise was minimized using the standard best practices (vibration isolation, environmental control), to ensure minimal electrical interference, a common electrical ground (to the KPFM ground) was provided. The recorded photodetector signal was then denoised using a two-step signal processing routine involving PCA followed by CWT analysis to accurately retrieve the *V*_CPD_ values. PCA is a well-established rank reduction technique that effectively converts the signal into a space of orthogonal basis vectors, preserving the complete signal information while facilitating noise separation.^40,62,63^ This rank reduction process transforms or decomposes the initially seemingly invariant and noisy photodetector signal (refer to Figure S2(A, B)) into a higher-dimensional space, wherein the noise-free data occupy a significantly smaller lower-dimensional subspace.^40,62,63^ We further utilize the singular value decomposition (SVD) method in conjunction with the information provided by the scree plot to calculate the number of principal components (PCs) to be retained (see Figure S2(C)).^63^ Further details on the PCA process are provided in the Methods section and Supporting Information (see Figures S3, S4). Utilizing this PCA-denoised data, the CWT-extracted amplitude scalograms (Figure 2(A, B)) clearly show the presence of applied bias pulses at the ω_AC_ frequency, thereby confirming the PCA is inherently useful to statistically clean the data set without any loss of spatiotemporal information. Further extraction of the amplitudes *A*_ω_ (at 15 kHz) and *A*_2ω_ (at 30 kHz) and phase φ_ω_ (at 15 kHz) allows the *V*_CPD_ calculations (eq 1). It should be noted that the DAQ, based on the multichannel acquisition conditions, imposes the 1 μs temporal resolution limit on the measurements (further details in the Methods section; see Figure S5(A–C)). Nevertheless, as observed, the calculated OL-WT-KPFM pulses (solid line labeled OL_SP in Figure 2(C, D)) effectively match the input signal, in both periodicity and absolute potential. In contrast, the *V*_CPD_ acquisition by CL-KPFM (dashed line labeled CL_SP in Figure 2(C, D)) is not as efficient. For instance, in the case of the 20 ms pulse period (Figure 2(C)), both the start and the end of pulses showed variations in the shape and amplitude of the detected pulse, highlighting the inability of the KPFM feedback loop to track the applied pulse. For the 2 ms pulse period (Figure 2(D)), while the OL-WT-KPFM was able to track the applied pulse (with an overall *V*_CPD_ error of <7%), the CL-KPFM, owing to the feedback and LIA time-constant limitations showed a *V*_CPD_ error of >70%. This arises from the condition of the pulse period being similar to the cantilever bandwidth (see Figure S6(A)). The observed triangular/sinusoidal shape of the signal arises from the closeness to the Nyquist frequency, undersampling, and the LIA bandwidth limitations, thereby leading to highly erroneous *V*_CPD_ values (see Figure S6(B)). In fact, across the measured pulse periods, the OL-WT-KPFM outperformed the CL-KPFM measurements (see Figure S7(A–H) for the scalograms and corresponding *V*_CPD_ graphs) in both the recovery of the applied pulse potentials and the corresponding rise times (*t*_r_) (see Figure S8(A, B)). From Figure S8(A, B), it can also be observed that the OL-WT-KPFM accuracy for the 20 ms pulse is slightly higher than for the 2 ms pulse. This can be explained from the perspective of comparable time scales of the pulse length to the cantilever’s response time (τ ∼ 1 ms), which prohibits the cantilever from reaching a steady state, thereby affecting the quantification accuracy (further explained in later sections).^64^ Nevertheless, considering these results, it can be concluded that the OL-WT-KPFM’s accuracy and temporal resolution for measuring the transients around and faster than the cantilever bandwidth far exceed the CL-KPFM.

**Figure 3:**
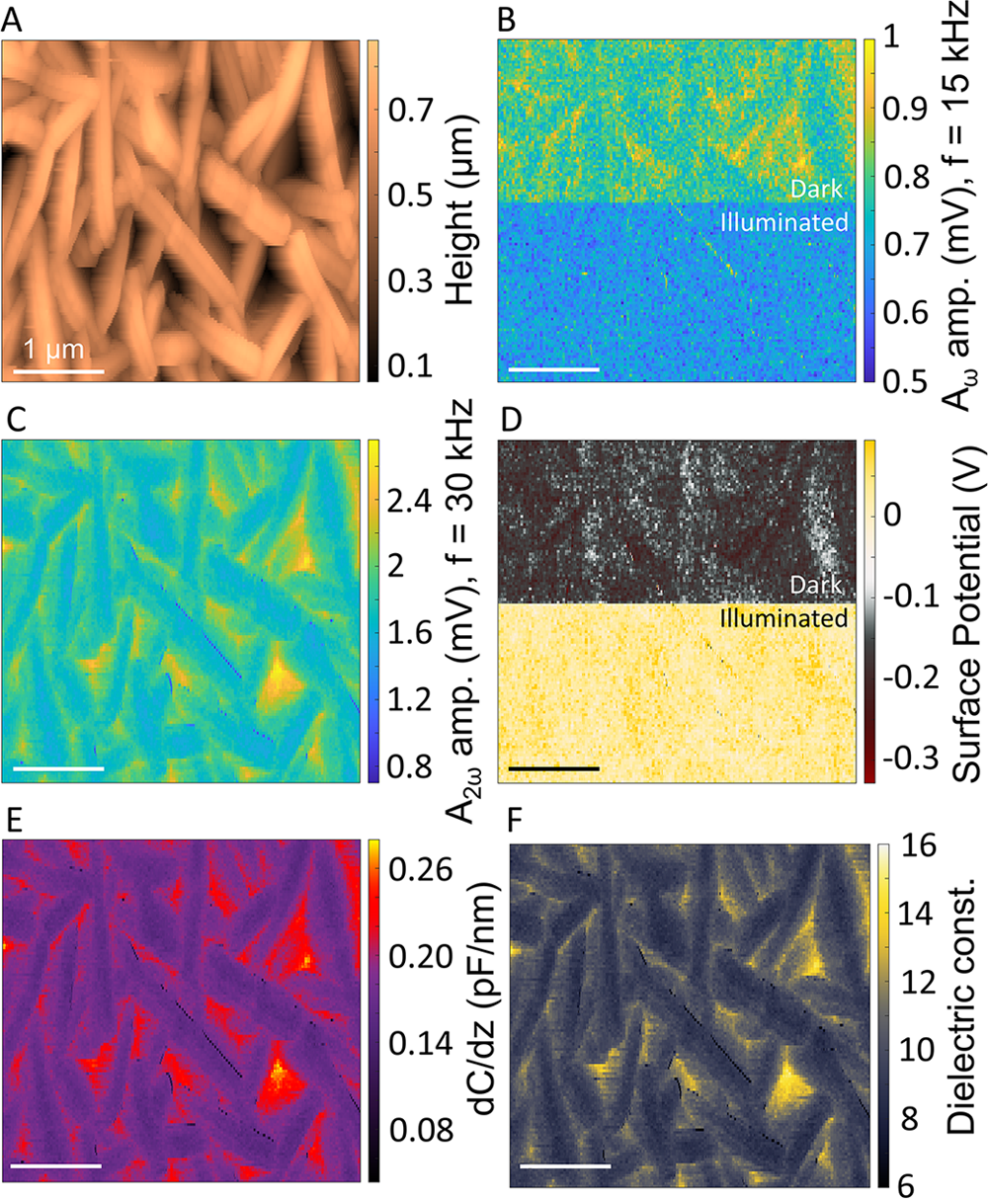
Imaging and information extraction capabilities of the OL-WT-KPFM technique. (A) Topography of the n-type BiOI sample. The CWT can extract the (B) first harmonic (*A*_ω_), (C) second harmonic (*A*_2ω_), and the corresponding (D) surface potential from the complete photodetector data stream. (E) The *∂C/∂z* mapping of the BiOI sample and the corresponding (F) dielectric constant (ε) values were computed for the sample. The dotted line in (B) represents the switching ON of the illumination to the sample. The horizontal scale bar represents 1 μm.

Besides the enhanced time resolution, we further tested the static and dynamic imaging capabilities of the OL-WT-KPFM method. Owing to its small band gap (1.8 eV), corresponding absorption in the visible spectrum, and a modifiable energy band structure, we have used BiOI to not only show the performance of our proposed technique to quantify the illumination-induced SP transient but also probe the dynamics of photogenerated charge diffusion. Unlike the other reported modalities, which require multiple LIAs to extract the physical properties, the OL-WT-KPFM allows the derivation of physical parameters without any additional hardware. Considering the entirety of the captured time-domain signal from one such 4 μm^2^ scan of BiOI, where besides the vertical-flake-like topography^65^ (Figure 3(A), which comes from the first pass), the CWT was able to *synchronously* reconstruct the SP variation (arising from the illumination effects, Figure 3(D)), capacitance gradient (Figure 3(E)), and the dielectric constant (Figure 3(F)) maps, respectively.^65^ Upon illumination of the sample (see the dotted line in Figure 3(B), corresponding to the switching ON of the light), the generation of the positive surface photovoltage (upswing of ∼196 mV between the dark and illuminated states) can be explained via the movement of the photogenerated charge carriers which are acted upon by the internal electric field and band bending.^65^ This ensures that the minority charge carriers (holes) diffuse to the surface of BiOI, while the electrons move toward the bulk (FTO). Further explanation and corresponding band diagram are provided in the Supporting Information and discussion around Figure 5(B), respectively. Similar to the pulse-bias measurements, the change of the *V*_CPD_ was reflected in the variation of the *A*_ω_ amplitude (Figure 3(B)) and the phase φ_ω_ (Figure S10) values. The *A*_ω_, *A*_2ω_, and φ_ω_ maps were utilized to create the SP image (Figure 3(D)) under dark and illuminated conditions, which showed high consistency with the CL-KPFM-derived SP map (see Figure S11). As the CWT extracts the amplitude and phase for the entire signal including the transient response at a 1 μs temporal resolution (DAQ limited; see Figure S5(A–C)), the non-averaged OL-WT-KPFM images carry a significantly greater deal of information on the surface charge-diffusion occurring in BiOI (see Figures S11, S12) than the time-averaged CL-KPFM.

**Figure 4:**
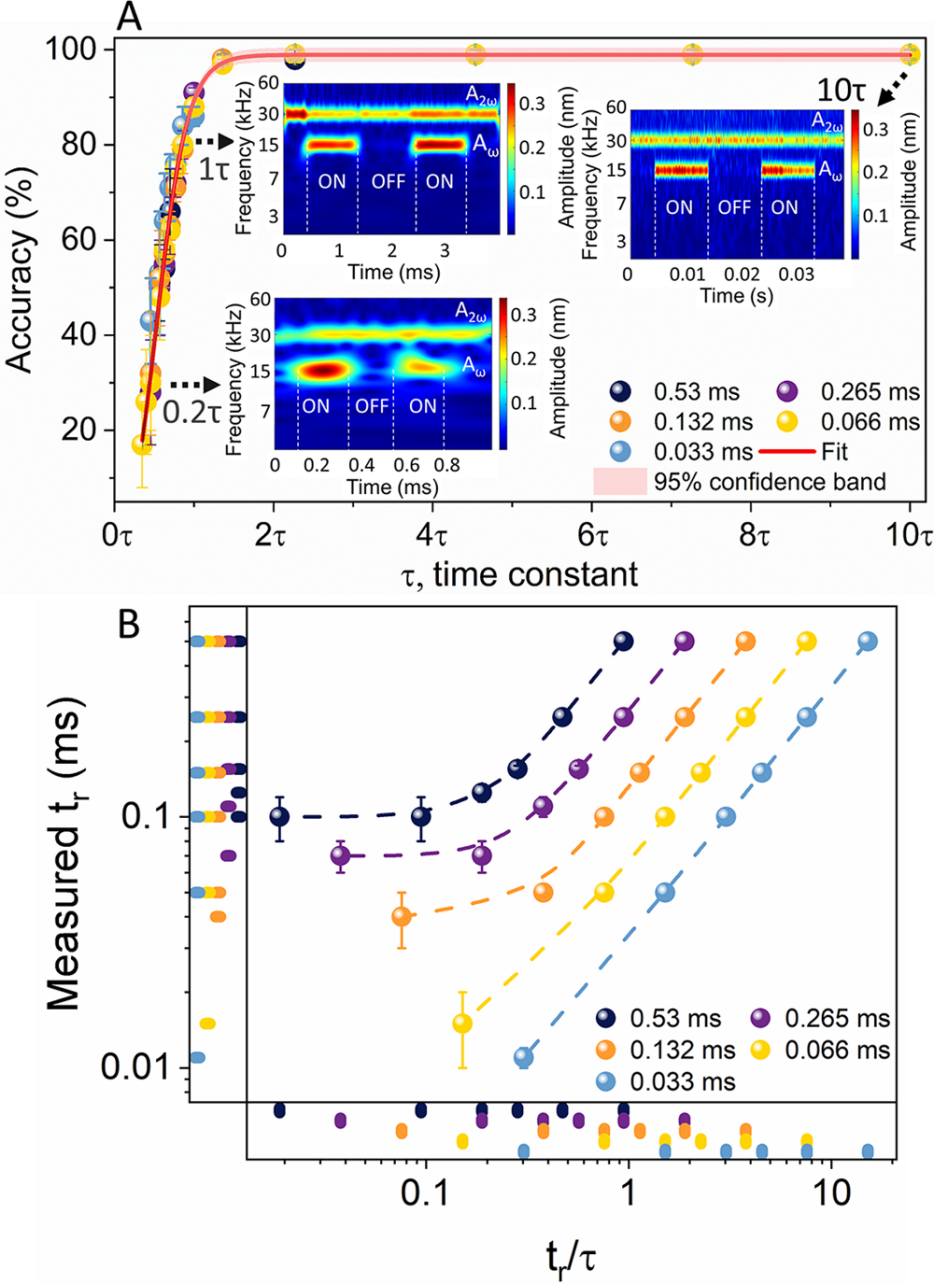
Numerical simulation of accuracy and transient detection capabilities of the proposed OL-WT-KPFM method. (A) Comparison of the accuracy of the detected transient pulses for the corresponding response times (τ) of different cantilevers. The inset scalograms (from experimentally detected pulses of 500 μs, 2 ms, and 20 ms pulse periods, corresponding to ∼0.2τ, 1τ, and 10τ, respectively) were generated via PCA filtering followed by the CWT analysis of the photodetector signal. (B) Comparison of the measured rise time of the various cantilevers to an applied step input. While the conventional 75 kHz cantilever can fully detect and quantify perturbations of ∼100 μs, the use of higher-frequency cantilevers will allow transient detection and quantification until ∼10 μs. Please note that the individual points on the *x-* and *y-*axis represent the corresponding data. The simulation parameters of the cantilever are *k* = 2.59, *Q* = 250, C′_*z*_ = 1 × 10^–9^, *V*_AC_ = 1.5 V, and *V*_CPD_ = 1 V. The natural resonant frequencies are ω_0_ = 75, 150, 300, 600, and 1200 kHz, respectively.

**Figure 5:**
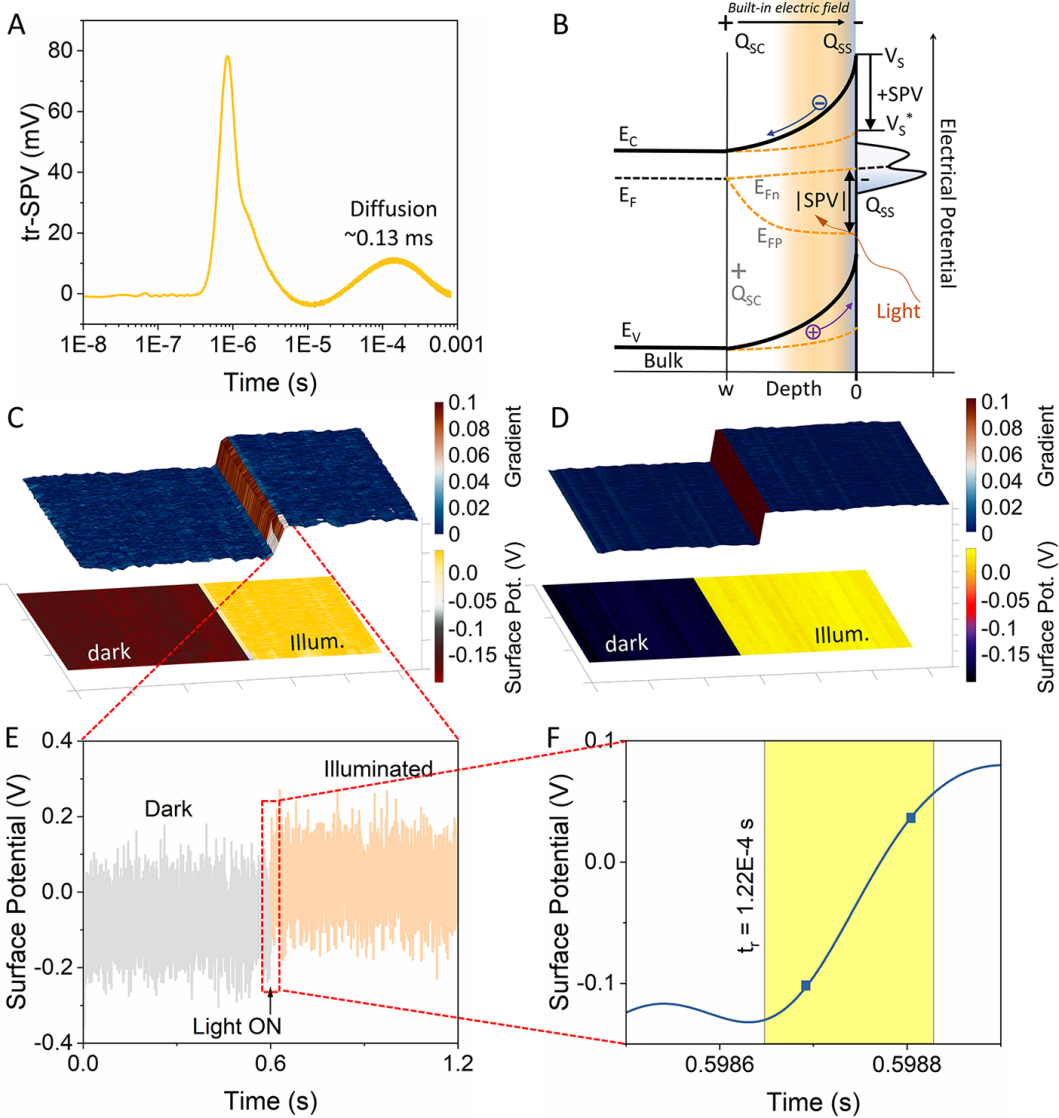
Bulk tr-SPV and single-pixel OL-WT-KPFM, CL-KPFM measurements on BiOI. (A) tr-SPV measurement of the BiOI sample highlighting the diffusion process and associated time regime, which is then subsequently measured in OL-WT-KPFM. (B) Schematic band diagram of an n*-*type semiconductor with a depletion layer (space charge region, SCR) and negative charge trapped at surface states in the dark (black lines) and under illumination (orange lines). *E*_C_, *E*_V_, *E*_F_, *E*_Fn_, *E*_Fp_, *Q*_SC_, *Q*_SS_, *V*_S,_ and *V*_S*_ denote the conduction and valence band edges, the Fermi energy in thermal equilibrium, the quasi-Fermi energies of electrons and holes under illumination, the uncompensated space charge, the charge in surface states, and the surface potential in the dark and under illumination, respectively. Reprinted (adapted) with permission from Chen, R.; Fan, F.; Dittrich, T.; Li, C. Imaging Photogenerated Charge Carriers on Surfaces and Interfaces of Photocatalysts with Surface Photovoltage Microscopy. *Chem. Soc. Rev.***2018**, 47 (22), 8238–8262. Copyright 2018 Royal Society of Chemistry. (C) The OL-WT-KPFM mapping with 1 μs temporal resolution allows the probing of the steady state SP response (see the 2D projection of the OL-WT-KPFM surface potential image in the lower panel) but can also probe (E) the switching ON transient (across the line, enclosed in the red lines in (C)) with an (F) extracted rise time of ∼112 μs, with a slope of 1.22 mV/μs. (D) The CL-KPFM measurements on a single pixel allow the extraction of change in the closed-loop SP profile upon switching ON and OFF the light source (see the projected 2D image in the lower panel) but prohibit the extraction of the transient response.

Based on the relationship of the capacitance gradient, *∂C/∂z* with the second harmonic (see eq 5, Methods section), quantitative measurements have been extracted (Figure 3(E)) via careful consideration of the *A*_2ω_ mapping (Figure 3(C)).^37^ The capacitance gradient (*∂C/∂z)* changes as the tip scans across the sample surface, capturing the variations in the tip–sample capacitance which have their origins in the topography and dielectric properties of the underlying material. A stronger *∂C/∂z* signal was observed corresponding to large changes in the topological features, while the BiOI flakes themselves showed a relatively constant value. Following the method proposed by Salomão et al.,^66^ we have used the sphere–plane model assuming a spherical tip to calibrate the proportionality constant, characteristic of the experimental setup. The subsequent dielectric constant mapping revealed a mean value of ∼10, which lies well within the range of 8–15 for BiOI, consistent with our earlier bulk measurements.^65^

Given the ability of the OL-WT-KPFM to probe the material properties at a 1 μs resolution, simulation-informed experiments were designed to enable quantifiable transient measurements. While the complete details of these simulations are provided in the Methods section and Supporting Information, below, we briefly discuss the important outcomes. Five different cantilevers with the same quality factor, *Q*, but different resonant frequencies, ω_0_, were simulated to cover varying response times (τ = 2*Q/ω*_0_) from 33 μs to 0.53 ms (see Figure 4(A)). It was observed that for continuous pulses the accuracy of the reconstructed SP increases exponentially as the pulse period increases, with over 90% accuracy achieved at a period around and above 0.9τ. The OL-WT-KPFMs’ detection ability was verified by comparing it to the experimental data (see inset of Figure 4(A)), wherein for continuous pulses shorter than the cantilever response time, τ, the accuracy of the SP calculation is reduced, as the cantilever is unable to reach the steady state. We have also simulated the case for a single transient in the form of an applied step input (Figure 4(B)) with varying rise times (*t*_r_) ranging from 0.01 to 0.5 ms to the aforementioned cantilevers of varying response times, τ. As seen in Figure 4(B) in the plot of *t*_r_/*τ* vs the measured rise time, while the conventional 75 kHz cantilever allows higher accuracy of quantification, albeit with a limit of detection of 90 ± 20 μs, for high-frequency cantilevers, the OL-WT-KPFM allowed fully quantifiable transient detection until 10 ± 1 μs. For the 1200 kHz cantilever, even faster transients can probably be detected and quantifiable; however, they have not been simulated in our work. It should be noted even that in the best-case scenario the G-mode requires one full period to analyze the signal^41,57^ and thus prohibits any transient measurement below this limit. In the case of the OL-WT-KPFM technique, while any transient equal or greater than one full period can be reliably and accurately detected, in the case where the transient is of subperiod scale, thanks to the WT’s ability, the OL-WT-KPFM technique is capable of detecting them. Thus, a higher temporal resolution can be achieved by using higher resonant frequency probes (with shorter response times) in conjunction with higher-speed DAQs. Similarly, by applying an AC drive frequency (ω_AC_) closer to the cantilever’s natural resonant frequency, the rise time of the detected pulse is lower, which means that the applied pulses can be detected faster, however, with reduced accuracy (see Figures S13, S14, and S15). We have further extracted the temporal resolution of the underlying WT method via the cantilever response to an applied chirp signal whose pulse periods were linearly swept from 0.1 ms to 200 ns over a period of 5 ms (Figure S16). The reconstructed force and displacement corresponding to the ON/OFF states could be distinguished for the entire measurement duration up to 200 ns, thereby providing the ultimate temporal resolution limit.

The high temporal resolution and ability to isolate signal from a significant noise floor (see Figure S17), therefore, allows the OL-WT-KPFM to investigate transient phenomena arising from the changes in the material’s electronic properties prompted by physical stimuli. To illustrate this, we demonstrate single-pixel, illumination-induced spectroscopic SPV measurements on BiOI.^26,65^ Prior to this, we carried out the bulk tr-SPV measurements to probe charge carrier dynamics by capturing events ranging from the generation/separation of electron–hole pairs to diffusion and recombination. As seen in Figure 5(A), upon illumination, the abrupt increase in tr-SPV arising from the early stage charge separation and drift is visible as the fast component, centered at 0.84 μs.^67^ Subsequently, a slow positive change in tr-SPV is observed in the range of 2–10 μs related to the holes’ slow drift velocity, and finally, the slow diffusion of charge carriers to the surface leads to the observed spectral component at 0.13 ms. While the CL-KPFM captures the diffusion-led corresponding change in the V_CPD_, it *cannot* capture the underlying transient itself. This paradigm, arising from the LIA time constant, holds true for both single-pixel and large-area measurements (Figure S9(A, B)) and thus immediately precludes the extraction of any transient response. As can be seen in the CL-KPFM measurements shown in Figure S1, KPFM captures the diffusion-led corresponding change in the *V*_CPD_, but it *cannot* capture the underlying transient itself. This paradigm, arising from the LIA time constant, holds true for both single-pixel and large-area measurements (Figure S9(A, B)) and thus immediately precludes the extraction of any transient response. As can be seen in the CL-KPFM measurements shown in Figure S9(C), the BiOI/FTO sample shows a strong SPV response. The average dark SP of BiOI was −0.175 V, which upon illumination dramatically rises to 0.027 V, providing an increase in the SPV value (SPV = (ϕ_sample_^*^ – ϕ_sample_)/*e* = *V*_CPD_^*^ – *V*_CPD_ = Δ*V*_CPD_) of ∼200 mV. It should be noted that this SPV response in semiconductors arises from the surface band bending which induces a space charge region (SCR)/depletion layer, leading to photogenerated e^–^–h^+^ pairs being separated by the built-in electric field in this region.^26^ In the case of n-type BiOI, this leads to the holes drifting toward the surface, while the electrons travel to the bulk, thus leading to an overall reduction of the surface charge (and SP) or surface band bending leading to positive SPV via the splitting of the quasi-Fermi levels *E*_FN_ and *E*_FP_, with the SPV being equal to the splitting potential (Figure 5(B)).^26^ However, as CL-KPFM provides a spatiotemporal averaged response of the sample to illumination, it is not suitable to investigate the temporal evolution of the phenomenon.

As demonstrated in Figure 5(C, D) showing the 2D projection of the SP (lower panels), the OL-WT-KPFM can probe the sub-ms evolution of the SPV (corresponding *A*_ω_, *A*_2ω_, and φ_ω_ maps in Figure S18) and captures the rapid rise in the potential upon the switching ON of the light. Please see the corresponding discussion in the Supporting Information and Figure S19 for an explanation of the conversion of captured photodetector signal to a 2D image. This rise time was calculated to be ∼112 μs (with a slope of 1.22 mV/μs; see Figure 5(E, F)) and compares well with the measured bulk tr-SPV transient behavior. Considering the gradient across the surface potential maps (see the top panels of Figure 5(C, D)), in the case of CL-KPFM this gradient was a constant, constrained by the feedback loop, while for OL-WT-KPFM, as the steady state was perturbed, the changes in the cantilever motion arising from the transient were fully captured. Furthermore, based on the time-averaged maps (shown in Figure S20) and corresponding histograms derived from them (shown in Figure S21), a wider distribution of the SP values can be observed from OL-WT-KPFM measurements compared to the LIA-derived CL-KPFM measurements. This essentially means that when undertaking the CWT analysis, all the transients and changes in accordance with the DAQ's sampling frequency, that the cantilever experiences, are preserved, and thus the OL-WT-KPFM technique is not only fully quantifiable but also displays high sensitivity. To eliminate the possibility of observing cantilever dynamics themselves, the cantilever ring-down time (τ_ring-down_ = *Q/*πω_0_) needs to be considered, which afflicts the OL-WT-KPFM in the same manner as the other fast transient KPFM modalities. For the electrically conducting probes utilized in this work (PPP-EFM and FMV-PT), the calculated mechanical bandwidth is ∼320 Hz with τ_ring-down_ ∼ 1 ms, respectively, and thus imposes the upper limit to accurately quantify continuously varying transients arising from repetitive pulses whose time scale is less than 0.25τ of the cantilever ring-down. Nevertheless, the biggest advantage of our proposed method is its ability to detect the single transient response arising from any perturbation in accordance with the DAQ's sampling frequency with a high accuracy, within the constraints of the bandwidth-imposed limits on the quantification accuracy. For instance, in the case of switching on dynamics of BiOI, as the transient occurs between two steady states, the transient is fully captured, and as the system reaches its steady state, the value of the SP calculated matches well with the closed-loop values (after 1 ms), thus making the OL-WT-KPFM measurements both fast and quantifiable. We have further carried out KPFM simulations for a 75 kHz cantilever by modeling the switching ON of the illumination as a bias pulse (Figures S22, S23). Based on these simulations in conjunction with the experimental evidence, it can be observed that a *V*_CPD_ error of typically around 5% is achieved, and the calculated rise time is consistent with the observed values. Therefore, the deployment of OL-WT-KPFM will allow investigation of the transient phenomena by combining the already established high lateral resolution of the technique with a temporal resolution, which has space for further improvement.

## Conclusions

In conclusion, using wavelet transform, we present a feedback-free, time-resolved KPFM technique capable of detecting the μs electrodynamics and accurately quantifying the sub-ms transients in the surface potential variations. The OL-WT-KPFM technique offers a significant pathway for undertaking fast and quantifiable dynamic measurements of nanoscale electronic behavior and multimodal characterization of materials. Unlike some of the other windowed Fourier techniques, which operate at a fixed resolution, the use of wavelets provides an adjustable time-frequency resolution. However, similar to other fast transient KPFM modalities, the OL-WT-KPFM is limited by the mechanical bandwidth and associated ring-down time of the cantilever as well as the DAQ's sampling rate. Nevertheless, the technique simultaneously maps the surface potential, capacitance gradient, and dielectric constant of the materials at a μs temporal resolution, in a single elegant experiment, without using multiple LIAs or external hardware. Furthermore, due to the inherent open-loop nature of WT-OL-KPFM, our method is capable of minimizing the convolution effects arising from the crosstalk typically observed in the closed-loop implementation. The use of wavelets and principal component analysis/singular value decomposition ensures that the noise present in the captured photodetector signal is minimized, allowing quantification of the surface potential with high accuracy and enhanced spatiotemporal resolution. It is expected that using high-speed DAQ and ultra-high-frequency cantilevers, the OL-WT-KPFM technique holds promise for detecting and quantifying the nanoscale charge dynamics and the sub-μs electron/hole transport for static and *in-operando* measurements alike.

## Methods

### Principal Component Analysis

PCA is a powerful tool in denoising AFM measurements.^62^ In this work, PCA is used as a pre-processing step for the analysis of the photodetector signal. PCA is essentially considered to separate the noise from the signal information by transforming the signal into a high-dimensional vector space which represents the data, while noiseless data occupy a small subspace with lower dimensionality.^63^ Further discussion is provided in the Supporting Information.

### Wavelet Transform Principle

Within the domain of signal processing, WT is a powerful tool for analyzing aperiodic, noisy, nonstationary, and transient signals. WT uses wave-like functions, which are defined as small oscillations with a quick decay, called wavelets, which are dilated and translated along the signal.^62^ WT further quantifies the local matching and correlation of the translated and dilated wavelets with the signal; hence, if the wavelet correlates well with the signal, a large value will be obtained for the WT. The transform values are calculated at various locations (relating to time) and at various scales (relating to the frequency) of the signal. Mathematically, WT can be defined as the convolution of the signal with the wave-like function called the “mother wavelet”. Continuous wavelet transform, described as the “mathematical microscope of data analysis”, is a type of WT that provides a high-resolution time–frequency representation of a signal. CWT is a sliding convolution of the signal, *x*(*t*), and the mother wavelet, Ψ(*t*), defined as eq 2:
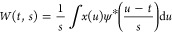
2where *s* and *t* are the scale and the time shift of the mother wavelet, Ψ, respectively, and Ψ* represents the complex conjugate of Ψ. *W*(*t*, *s*) represents the wavelet coefficient of the signal localized in (*t*, *s*), called the “daughter wavelet”. As denoted, CWT uses time–scale analysis as an alternative to time–frequency. Accordingly, CWT transforms the signal into each scale by using a band-pass filter localized in ω_*s*_ frequency, while all scales have a constant relative frequency of Δω_*s*_*/*ω_*s*_. It is worth noting that CWT, like all signal analysis tools, suffers from the limitation to make an adjustment between time and frequency resolution. However, CWT follows the Heisenberg principle, which states that the product of uncertainty in time and frequency should be more than or equal to a constant value.^68^ CWT benefits from using varying time–length operators (mother wavelets) to enhance the time–frequency localization. This allows the use of long wavelets in analyzing lower frequency components of the signal to improve frequency localization and, conversely, shorter wavelets in analyzing higher frequency components of the signal to allow for high time–localization. Accordingly, CWT is able to capture the instantaneous amplitude and hence the transient of a nonstationary signal component in the time domain. However, CWT suffers from edge-effect artifacts, which affect the calculation of instantaneous amplitude at the beginning and at the end of the time-domain signal. This arises from the convolution of the mother wavelet with the signal in areas where the mother wavelet length exceeds the signal length. In this study, we have used generalized Morse wavelets (GMWs) as the mother wavelet in implementing the CWT analysis. GMWs are the recommended superfamily of wavelets for analyzing modulated signals since they allow for preserving a wide range of signal characteristics while remaining analytical.^69^ For the mother wavelet, we have used a symmetry parameter, γ = 3, to have zero skewness and to ensure minimum Heisenberg-like effects, and the time–bandwidth product of *P*_*β,γ*_^2^ = 60.^70^ The time–bandwidth product itself can be defined as *P*_*β,γ*_^2^ = β·γ where β denotes the compactness parameter of the mother wavelet.

In order to have a reliable analysis, and to overcome the edge effects at the beginning of the signal, the wavelet footprint parameter  was used to inform the minimum duration required to detect 95% of the energy of the frequency of scale *s*, ω_*s*_.^70^ Clearly, by capturing more oscillations than the footprint length, the wavelet limitation concerning the edge effect can be overcome and WT can reliably measure the instantaneous amplitude and phase of the captured photodetector signal continuously for every data point while detecting any transients and perturbations. For higher frequencies, as understood from the equation above, a shorter footprint duration is required. It should be noted that in this study all the biases/pulses on the signal were applied when the footprint length was exceeded.

Considering the use of GMWs as mother wavelets in CWT analysis, the amplitude of the daughter wavelet corresponding to a particular scale in the time domain can be calculated as the magnitude of the complex CWT coefficient as defined in eq 3:
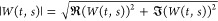
3where  and  present the real and imaginary parts of the complex CWT coefficient, respectively. Theoretically, by applying eqs 2 and 3, the amplitude of the higher harmonics of the photodetector signal can be calculated. However, due to the deficiencies of dilated GMW filters, the amplitude of the second harmonic may show fluctuations.^69^ To minimize these fluctuations, discrete wavelet transform (DWT) is applied to the magnitude of the daughter wavelets in the desired scale. DWT is a type of WT transform performed in discrete steps. In this study, for the analysis of the second harmonic, a high-order Daubechies wavelet (db45) is chosen as the mother wavelet, since the second harmonic of the photodetector signal is assumed to be smooth and sinusoidal.^71^ Therefore, the corresponding daughter wavelet is decomposed using DWT, db45 mother wavelet, up to 9 levels. The approximation is then used as a second-harmonic amplitude. Further discussion on the magnitude response of this filter is provided in the Supporting Information (also see Table S1 and Figure S24). Moreover, cross wavelet transform (XWT) allows for the analysis of two signals, simultaneously, in time and frequency domains. Accordingly, by applying XWT to the photodetector and the AC drive signal and quantifying the interaction between them, the local relative phase of the photodetector signal is calculated (further discussion is provided in the Supporting Information).

## Experimental Section

### Synthesis of BiOI

The growth of photoactive n*-*type bismuth oxyiodide nanoflakes on fluorine-doped tin oxide (FTO) substrates was carried out using an acidic-medium, potentiostatic electrochemical method. While complete details are provided in our earlier work, the synthesis can be summarized as follows.^65^ Bismuth(III) nitrate pentahydrate (Bi(NO_3_)_3_·5H_2_O), ≥98.0%), potassium iodide (KI, ≥99.0%), nitric acid (HNO_3_, ≥70%), and *p*-benzoquinone (≥98.0%) were purchased from Sigma-Aldrich (UK) and used as obtained without any further purification. A solution of 0.04 M Bi(NO_3_)_3_ was prepared by dissolving Bi(NO_3_)_3_·5H_2_O in 25 mL of 0.4 M KI solution (pH = 1.7, via the addition of HNO_3_) and added to 10 mL of 0.23 M *p*-benzoquinone absolute ethanol solution. For electrodeposition, a standard three-electrode configuration was utilized with a masked FTO (resistivity ∼7 Ω/sq.) as the working electrode, Ag/AgCl as the reference electrode, and Pt as the counter electrode, respectively. The potentiostatic deposition was carried out at −0.3 V vs Ag/AgCl for 60 s to obtain BiOI nanoflake films. The as-prepared samples were further rinsed with DI water and further annealed in a muffle furnace (in air) at 350 °C for 2 h to ensure high crystallinity.

### Numerical Simulations

Numerical simulations of the cantilever motion, governed by the following equation, were performed using a fourth-order Runge–Kutta algorithm in C++:
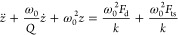
4where *z*, *F*_d_, ω_0_, *Q*, and *k* are the tip deflection, drive force, natural resonant frequency, quality factor, and spring constant of the cantilever, respectively. The *Q* determines how the cantilever relaxes to equilibrium and, in practice, determines the cantilever response time (inverse of the mechanical bandwidth of the cantilever) as τ = 2*Q*/ω_0_. The external force on the right-hand side of eq 4 consists of two terms: time-dependent excitation force, *F*_d_*= F*_0_ sin(ω*t*), where ω is the excitation frequency and displacement-dependent interaction force between the cantilever-tip ensemble and sample, *F*_ts_. The details of parameters used in the simulation are given in Figure 4(A, B) and the Results and Discussion section.

### KPFM Measurements

Both the conventional CL-KPFM and proposed WT-OL-KPFM measurements were carried out using commercial AFM systems, Asylum Research MFP-Infinity and a DI3100, Digital Instruments, (now Bruker Corporation), provided with a signal access module (SAMIII, Digital Instruments). The photodetector signal was recorded via a data acquisition board (NI USB-6366, 16-bit, 2 MS/s (for single channel)/1 MS/s (for multiple channel acquisition), National Instruments). Platinum-coated probes, FMV-PT (Bruker) and Nanosensors PPP-EFM (both with nominal *k* (2.8 N/m) and a nominal ω_0_ of 75 kHz), were employed for the experiments, and Sader’s method was used for the evaluation of *k* and the *Q*-factor.^72^ An electrical KPFM-EFM calibration sample (Budget Sensors) comprising an oxide-covered Si substrate with arrays of Au and Al lines (4–40 μm pitch) connected to Au and Al pads, respectively, was used for the pulse experiments by connecting the sample to an arbitrary function generator (AFG, MDO3014, Tektronix).

The DAQ-captured time-domain signal was run through the developed MATLAB code to extract the first- and second-harmonic gains as described by the following equations:^37^
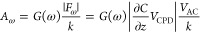
5
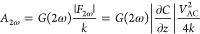
6where *G*(ω) and *G*(2ω) are the cantilever transfer function gains at the two frequencies, obtained from
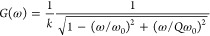
7For the CL-KPFM measurements, standard two-pass experiments were carried out, with feedback during the lift pass applying DC voltage to the probe to match the *V*_CPD_, while for the WT-based OL-KPFM, the lift pass feedback was disabled. Data for pulse measurements were acquired at a single pixel in the point scan configuration with a 0 nm scan size. These 0 nm scans were typically carried out at a scan rate of 0.5–2 Hz with 256 points/line and 256 scan points, unless stated otherwise. Point scans were also utilized to elucidate the effect of choice of lift height and ω_AC_ (Figure S25). Photostimulation-based surface photovoltage experiments were performed on the annealed BiOI samples, by illuminating the sample from a white light LED with a cutoff wavelength of 400 nm and focused using a 20× objective lens with an overall spot size of ∼300 μm.

### tr-SPV Measurements

The time-resolved surface photovoltage spectra (tr-SPV) were conducted on a transient surface photovoltage spectrometer (CEL TPV2000, Beijing China Education Au-light Co., Ltd., Beijing, China) using an Nd:YAG pulsed nanosecond laser (355 nm@60 mJ, 20 Hz), with a time resolution of 5 ns.

## Supporting Information Available

Background information on CL-KPFM, PCA, DWT, and WT phase analysis; influence of experimental parameters on OL-WT-KPFM measurements; temporal resolution of WT and effect of noise on OL-WT-KPFM measurements; SEM and EDX analysis of KPFM calibration sample; captured photodetector signal and corresponding scree plot for PCs; PCA-led denoising of the raw photodetector signal; error quantification for raw and PCA-denoised signal; CL-KPFM pulse data and error values; CWT-derived amplitude scalograms and recovered SP values; accuracy of CL-KPFM vs OL-WT-KPFM methods; CL-KPFM-based imaging of BiOI; OL-WT-KPFM extracted phase data; comparison of CL-KPFM- and OL-WT-KPFM-derived SP maps; spatiotemporal evolution of the surface photovoltage of BiOI; numerical simulation of the accuracy of the proposed OL-WT-KPFM method; simulation of a 300 and 1200 kHz cantilever subjected to varying pulses; simulation results of the calculated SP and rise time; time resolution of the WT technique; effect of noise on CPD extraction via WT; extraction of harmonics and phase from photodetector data stream; CWT-derived SP maps for single-pixel BiOI transient; histograms of the CWT-derived SP amplitudes; simulations for transient response time quantification at ω_AC_ = 25 and 10 kHz. Magnitude response of DWT; lift height- and ω_AC_-dependence of measured surface potential; table of relative error of the second-harmonic amplitude (PDF)
