# Supplementary material for: Unraveling Spatiotemporal Transient Dynamics at the Nanoscale via Wavelet Transform-Based Kelvin Probe Force Microscopy
Source: ACS Nano. 2023 Oct 25;17(21):21506–17. doi: 10.1021/acsnano.3c06488 (PMC10655243; doi:10.1021/acsnano.3c06488)
Supplement: Supplementary file 1 — nn3c06488_si_001.pdf [file nn3c06488_si_001.pdf]

Supporting Information

Unravelling Spatio-temporal Transient Dynamics at Nanoscale *via* Wavelet Transform-based Kelvin Probe Force Microscopy

Pardis Biglarbeigi,[†,ǂ] Alessio Morelli,[†,‡] Serene Pauly,[§,‡] Zidong Yu,[ǁ] Wenjun Jiang,[⊥] Surbhi Sharma,[ζ] Dewar Finlay,[†] Amit Kumar,[§,‡] Navneet Soin,[†,] and Amir Farokh Payam[†,*]*

[†]Nanotechnology and Integrated Bio-Engineering Centre (NIBEC), School of Engineering, Ulster University, York Street, Belfast BT15 1AP, Co. Antrim, Northern Ireland, United Kingdom

[ǂ]School of Science and Engineering, University of Dundee, Nethergate, Dundee, DD1 4NH, Scotland, United Kingdom

[§]School of Mathematics and Physics, Queen's University Belfast, University Road, Belfast BT7 1NN, Northern Ireland, United Kingdom

[ǁ]Institute for Materials Research and Innovation (IMRI), University of Bolton, Deane Road, Bolton BL3 5AB, United Kingdom

[⊥]College of Transportation Engineering, Dalian Maritime University, Dalian 116026, China

[ζ]Centre for New Energy Transition Research Technologies (CfNETR), Federation University Australia, Churchill, Victoria 3810, Australia

[1]School of Science, Computing and Engineering Technologies, Swinburne University of Technology, P.O. Box 218, Hawthorn, Victoria 3122, Australia

‡*These authors contributed equally.*

Email*: n.soin@ulster.ac.uk; nsoin@swin.edu.au (N. Soin) & a.farokh-payam@ulster.ac.uk (A. F. Payam)*

This PDF file includes:

Supplementary Text
Figs. **S1** to **S25**
Table **S1**

Background Information

CL-KPFM principle

As discussed in the introduction, the CL-KPFM analysis relies on the measurement of the long-range electrostatic tip-sample forces, $F_{el} = -\frac{1}{2}\frac{\partial C}{\partial z}V^2$, which are predominantly capacitive in nature and where C and V are the capacitance and total voltage between the tip and the sample, and $\frac{\partial C}{\partial z}$ represents the capacitive gradient, respectively.[1] The KPFM controller then applies an AC voltage, V_{AC}, and a dc voltage, V_{DC}, between the sample and the tip, so that the total voltage under consideration of the V_{CPD} is given by:

$$F_{el} = -\frac{1}{2}\frac{\partial C}{\partial z}[V_{DC} - V_{CPD} + V_{AC}sin(\omega_{AC}t)]^2 \qquad \text{Eqn. S1}$$

where V_{CPD} is the contact potential difference, and ω_{AC} is the electrical drive frequency, respectively. Considering the expansion of Eqn. S1, it can be split up into static DC capacitive force (F_{dc}) and oscillatory forces at excitation (F_{ω}) and harmonic ($F_{2\omega}$) frequency components, as shown in Eqn. S2.

$$F_{el} = -\frac{\partial C}{\partial z}\left[\underbrace{\frac{1}{2}(V_{DC} - V_{CPD})^2 + \frac{1}{4}V_{AC}^2}_{F_{dc}} + \underbrace{(V_{DC} - V_{CPD})V_{AC}sin(\omega_{AC}t)}_{F_{\omega}} - \underbrace{\frac{1}{4}[V_{AC}^2 cos(2\omega_{AC}t)]}_{F_{2\omega}}\right]$$

Eqn. S2

The periodic electrostatic force leads to oscillations in the cantilever, whose amplitude (A_{ω}, $A_{2\omega}$) and phase (φ_{ω}, $\varphi_{2\omega}$) response is detected using an LIA at the applied bias frequency, ω_{AC}. The first harmonic amplitude, $A_{\omega} = -\frac{\partial C}{\partial z}(V_{DC} - V_{CPD})V_{AC}G(\omega)$, proportional to the transfer function $G(\omega)$ of the cantilever, is supplied to a feedback loop that continuously adjusts the DC bias, leading to the condition $V_{DC} = V_{CPD}$ and elimination of cantilever oscillations thus allowing for potential mapping of the sample.[1] Despite avoiding the issues related to the feedback operation,

retrieval of the amplitude and phase of the cantilever motion *via* the classical lock-in method still suffers from limitations in time resolution, which we propose to eliminate *via* the open-loop WT method.

Principal Component Analysis (PCA)

PCA is a statistical methodology that converts the observations into a set of uncorrelated orthogonal variables, called Principal Components (PCs). As suggested by Collins *et al.,* we reshaped the photodetector raw signal into an $n \times p$ matrix of A, where n shows the number of datapoints, and p represents the number of variables.[2] The variables are chosen considering the nature of the data, *e.g.,* each pulse is considered to be one variable. Accordingly, the number of datapoints for each variable can be obtained by the chosen variable itself, *e.g.,* the length of the pulse determines the number of datapoints in the variable. Matrix A was centered by subtracting the mean of each variable. Then the Singular Value Decomposition (SVD) was performed to calculate the PCs. SVD of matrix A can be defined as:

$$A = U \cdot S \cdot V' \qquad \text{Eqn. S3}$$

where U and V represent the left and right singular vectors, respectively, the diagonal matrix of S shows the singular values which are related to eigen values of covariance matrix $U,$ and S denotes the PCs, respectively. In order to reconstruct the noiseless signal, a few initial PCs will be chosen that retain the most amount of information, and the rest are excluded from the analysis. The number of these PCs can be defined by screening the scree plot of the singular values (or the eigen values) in descending order. The scree test suggests that the number of PCs is selected by the "elbow" of the curve, or the number of PC corresponding to the singular value that shows a clear separation between the steep slope of initial PCs and a gentler slope of the excluded PCs.[3]

For instance, in the case of 10 ms pulse length data, there were 481 pulses captured in the signal recording (see Fig. S2 (a, b). We have utilised each of these pulses as one variable *i.e.,* a total of 481 PCs and thus 481 singular values were obtained corresponding to each of these PCs. Based on the scree plot shown in Fig. S2(c), we have chosen the first five PCs which represent information with the highest amount of signal information beyond which the singular values plateau out – indicative of noise components, and thus act as the cut-off when denoising the data. The raw photodetector signal showed significant fluctuations (Fig. S3(a, b)) which upon PCA-denoising were significantly removed (Fig. S3(c, d)).

Discrete Wavelet Transform (DWT)

As discussed in the manuscript, the second harmonic obtained from the CWT time-frequency analysis is further filtered using a high-order Daubechies wavelet (db45), to minimise the fluctuations associated with the poor GMW filters. After decomposition of the daughter wavelet using db45, up to 9 levels, the approximation, lowpass filter, is then used as second harmonic amplitude. The magnitude response of the 9th level approximation (A9) of db45 filter is shown in Fig. S24.[4] As can be seen in Fig. S24, the magnitude response for f = 30 kHz for this DWT filter has a magnitude of 1.46e-15 dB, which can be considered negligible, showing that this filter can give reasonable accuracy in reporting the second harmonic amplitude. We have further simulated KPFM signals and analysed the magnitude of its second harmonic considering different SNRs. Table T1 shows the percentage of relative error with the simulated second harmonic amplitude.

WT phase analysis

Cross Wavelet Transform (XWT) is applied to the photodetector signal and the AC drive signal to calculate the interaction between the two time-series. XWT is an extension to WT that gives a measure of correlation and the link between two signals. XWT can find the local relative phase between the two signals[5] and is calculated as Eqn. S4:

$$W_{xy}(t,s) = W_x(t,s) \times W_y^*(t,s) \qquad \text{Eqn. S4}$$

where x and y are the two time-series signals (*i.e.,* cantilever and the drive signal), W is the CWT coefficient, or daughter wavelet, of the two signals localised at time t and scale s and W^* is the conjugate of the daughter wavelet. $W_{xy}(t,s)$ shows the local link between the two signals and the local phase can be calculated by Eqn. S5.

$$\angle W_{xy}(t,s) = arctan\,(\frac{\Im(W_{xy}(t,s))}{\Re(W_{xy}(t,s))}) \qquad \text{Eqn. S5}$$

The code for OL-WT-KPFM was written in MATLAB, which imports the captured raw photodetector and the AC drive signals (*.tdms format). Initially, a pre-processing procedure is applied on the photodetector signal to minimise the noise, through PCA. Consequently, using the first and second harmonic amplitude *via* Eqn 2. (main manuscript) and phase obtained through Eqns. S4 & S5, the V_{CPD} is calculated *via* Eqn. 1 (main manuscript).

Influence of experimental parameters on OL-WT-KPFM measurements

Influence of choice of ω_{AC}

To probe the underlying physics and the influence of experimental parameters on the time-resolved measurements, we have performed a range of simulations to understand and evaluate (i) the cantilever response time (τ) dependence of the recorded response, (ii) the effect of cantilever response time on the extracted transient SP, and finally (iii) the accuracy of transient detection. In

our simulations, we have solved the KPFM model (Eqn. 4, methods sections) using a fourth-order Runge-Kutta algorithm in C++. To model the KPFM response to pulse voltages as an applied electrostatic force, we have applied a single-frequency sinusoidal wave voltage at frequencies lower than the natural frequency of a cantilever (ω_0) superimposed with a DC square wave with different periods. Further details of the simulations are given in the materials and methods section. Five different cantilevers with different natural resonant frequencies (ω_0), albeit with the same Q factor, were selected. This covered the cantilever response time ($\tau = 2Q/\omega_0$) from 66 μs to 1.1 ms. The square wave periods were varied from 0.3τ to 10τ, with the resulting simulated SP provided in Fig. 4(a). As can be observed, there is an exponential growth in the accuracy of the reconstructed SP as the pulse period increases. In addition, for a period around and higher than 0.9τ, our approach has more than 90% accuracy in reconstructing the SP. Thus, by employing cantilevers with small τ, the proposed method can not only *detect* but also *accurately* quantify the -μs temporal range for the material's transient phenomena. For instance, using a 1.2 MHz cantilever ($\tau = 66\mu s$), the transient dynamics in SP of $60\ \mu s$ can be detected and quantified accurately *without any influence from the cantilever response*.

The detection ability of the proposed method is verified by comparison with the experimental data (insets in Fig. 4(a)). For pulses with a period shorter than the cantilever response time, although the method is capable of detecting the transient phenomenon, the SP value calculation accuracy is reduced, owing to the fact that the cantilever cannot reach its steady-state response; while for periods longer than the cantilever response time both detection and accuracy can be achieved. Thus, by appropriate probe selection, OL-WT-KPFM holds promise for outperforming previously reported methods, by excluding the effect of the cantilever transient. Fig. S13(a) shows the calculated SP for three different pulse periods when simulated for a

cantilever with a ω_0 of $75\ kHz$ and $\tau = 1.1\ ms$ (corresponding to the FMV-PT/PPP-EFM cantilever used for the CL- and OL-WT-KPFM measurements). The simulations confirm that for a pulse period longer than the response time of the cantilever, the SP can be reconstructed accurately, while with a reduction in pulse period (especially below τ) the accuracy of the quantification is affected, albeit the pulses can still be detected. Finally, to investigate the effect of AC drive signal frequency on the SP calculation, another set of simulations has been performed. Utilising the same cantilever as used in our experiments, we vary the AC drive signal frequency (ω_{AC}) from 7.5 kHz to 20 kHz to explore the relationship between the drive frequency, rise time, and the peak of the reconstructed SP pulses (see Fig. S13(a, b)). It can be summarised that with the application of an AC drive signal closer to the cantilever's natural resonant frequency, the rise time of the detected pulse is lower which means that the pulses can be detected faster, with, however, reduced accuracy. Therefore, for fast and accurate transient sensing, an optimal frequency for the AC drive signal needs to be identified, with these simulations providing the necessary framework for it. For instance, for the OL-WT-KPFM measurements shown in Fig. 2 (main manuscript), we utilised ω_{AC} = 15 kHz which provides an accuracy of 93.5% at a fast rise time of ~0.215 ms. Simulation results for various other cantilevers are provided (Fig. S13, S14, S15), underpinning the ability of the OL-WT-KPFM method to detect and quantify the -*ms* and sub-μs transients.

Influence of lift-height

For assessing the lift-height dependence of ω_{AC} on the A_ω and $A_{2\omega}$ harmonics, we have considered single pixel measurements utilising three different applied ω_{AC} values of 15, 20 and 25 kHz (keeping the V_{AC} constant at 3 V) and varying the lift height from 50 – 2000 nm, respectively. Similar to the measurements shown in Figs. 1, 2 of the main manuscript, a pseudo potential contrast

was created using a unipolar bias pulse applied to the Au pad, and as required by the OL-WT-KPFM technique, the applied bias pulse, drive signal and the cantilever deflection signals were simultaneously recorded using the DAQ and then further processed. The resulting CWT-derived scalograms (Fig. S25(a-i)) highlight the variation in measured oscillation amplitudes as a function of lift height and the applied ω_{AC}.

It can be clearly observed that keeping the lift height constant, a lowering of ω_{AC} led to a reduction in the amplitudes of both the A_{ω} and $A_{2\omega}$ harmonics (see Fig. S25(J, K)). Similarly, at a fixed ω_{AC}, an increase in the lift height again led to a reduction in the A_{ω} and $A_{2\omega}$ harmonics' amplitude with a clear asymptotic behaviour (Fig. S25(J, K)). Moreover, as compared to the behaviour of A_{ω}, the reduction in $A_{2\omega}$ was more pronounced (see the arrow marked in Fig. S25(K)) which could arise from the V_{AC}^2 dependence of $A_{2\omega}$. In an earlier work, Liscio et al.[6] have reported that the tip-sample distance can be expressed as: $d(t) = d_{AFM} + lift\ height + d_{osc} \sin(\omega t)$, where d_{AFM} represents the tip-sample distance during the topographic scan, and d_{osc} is the amplitude of the cantilever oscillation due to the V_{AC} bias. This distance *d(t)* oscillates around its mean value, which is constant during KPFM scan, however, d_{osc} (equivalent to A_{ω} in our case) decreases when the tip moves far from the surface due to a reduction in the tip-sample interactive forces (see Fig. S25(J)). Thus, irrespective of the nature of the KPFM measurements whether open- or (closed-loop), the harmonics (oscillation) amplitude shows a reduction with the increase in the lift height and the lowering of drive frequency ω_{AC}. Accordingly, the SP at significantly larger lift heights shows a pronounced variation and associated noise (see Fig. S25(L)) arising from increased tip-sample electrostatic force interaction volume,[7] i.e., tip convolution effect,[8] and a lower minimum detection potential owing to lower sensitivity[9]. It is therefore recommended to keep the OL-WT-KPFM lift-height in the nominal usual range of the conventional CL-KPFM measurements.

Temporal resolution of WT and effect of noise on OL-WT-KPFM measurements

To measure the temporal resolution of the WT method itself and the accuracy of the OL-WT-KPFM in the presence of noise – key factors dictating the transient measurements, further simulations were carried out. Based on the technique of Collins *et al.*,[2] the temporal resolution simulations utilised a chirp signal whose pulse period was linearly swept from 0.1 ms to 200 ns, over a period of 5 ms. We then extract the corresponding displacement of the cantilever (by WT) in response to this chirp signal. As can be seen in (see Fig. S16), in response to the applied pulse, the cantilever displays its maximum response at resonance, while the recovered displacement detected by the WT closely follows the applied pulse with its overall magnitude remaining constant. In fact, the reconstructed force and displacement corresponding to the ON and OFF states could be distinguished for the entire duration of the measurement until 200 ns (see Fig. S16). It should be noted that this time resolution of 200 ns (limited by the sampling frequency and associated Nyquist criteria) is ~3 orders of magnitude faster than the 9 μs reported by Collins *et al.*[2] and nearly 5 orders higher than the corresponding CL-KPFM measurements. To evaluate the accuracy of OL-WT-KPFM in the presence of noise, we have further simulated the KPFM model by adding white Gaussian noise to the signal with varying magnitudes of signal-to-noise ratio (SNR) ranging from 1000/1 (60 dB) to 1/10 (-20 dB). Unlike the typical FFT-based techniques which prohibit the extraction of accurate temporal information owing to aliasing, our simulations show that the WT can extract V_{CPD} values accurately across the 6.0 to -20.0 dB SNR range with less than 10% error (see Fig. S17).

Methodology for converting raw photodetector signal to 2D images

In our experiments, the photodetector and drive signal are collected by a DAQ connected to the cantilever. These signals are sampled at 1MHz and are gathered continuously for the full probing period, hence presented as a large 1D time series dataset for each sample. For the purpose of our analysis, the 1D signals are segmented using the scan rate where each segment represents one line of the scan and consequently one line of the measured image. Acknowledging that in KPFM measurements each line contains the tapping and the lift mode, the tapping segment is discarded and the amplitudes $(A_{\omega}, A_{2\omega})$, and phase (φ_{ω}) and consequently V_{CPD} of each line in lift mode are calculated by applying the proposed OL-WT-KPFM methodology. Accordingly, the images are produced by concatenating the results of all the lines, see Fig S19.

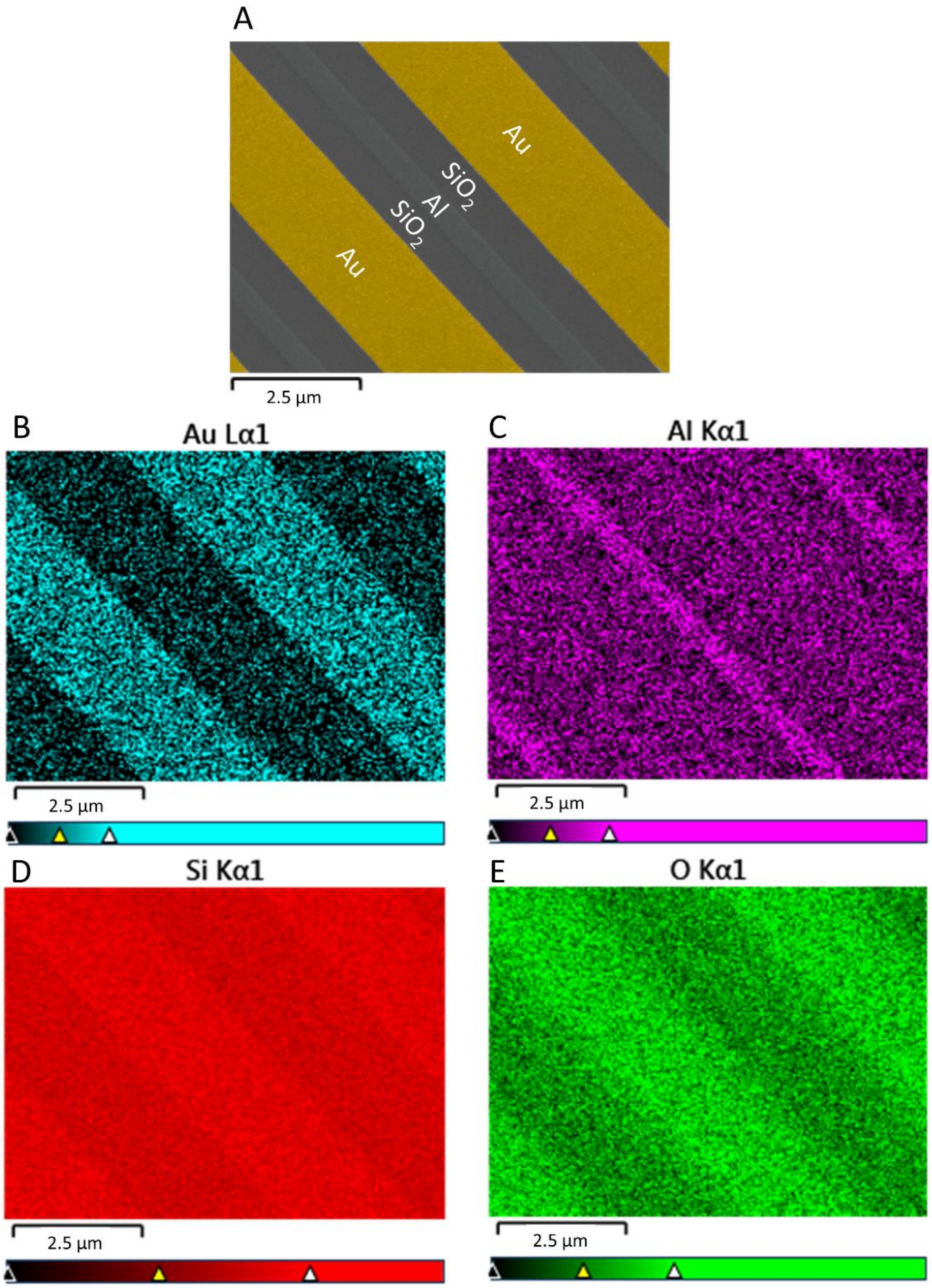

Fig. S1. SEM and EDX analysis of KPFM calibration sample. (A) False colored SEM image of the KPFM calibration sample highlighting the Au and Al wires on the SiO_2 substrate, derived using the SEM-EDX elemental mapping of (B) Au, (C) Al, (D) Si, and (E) O, respectively.

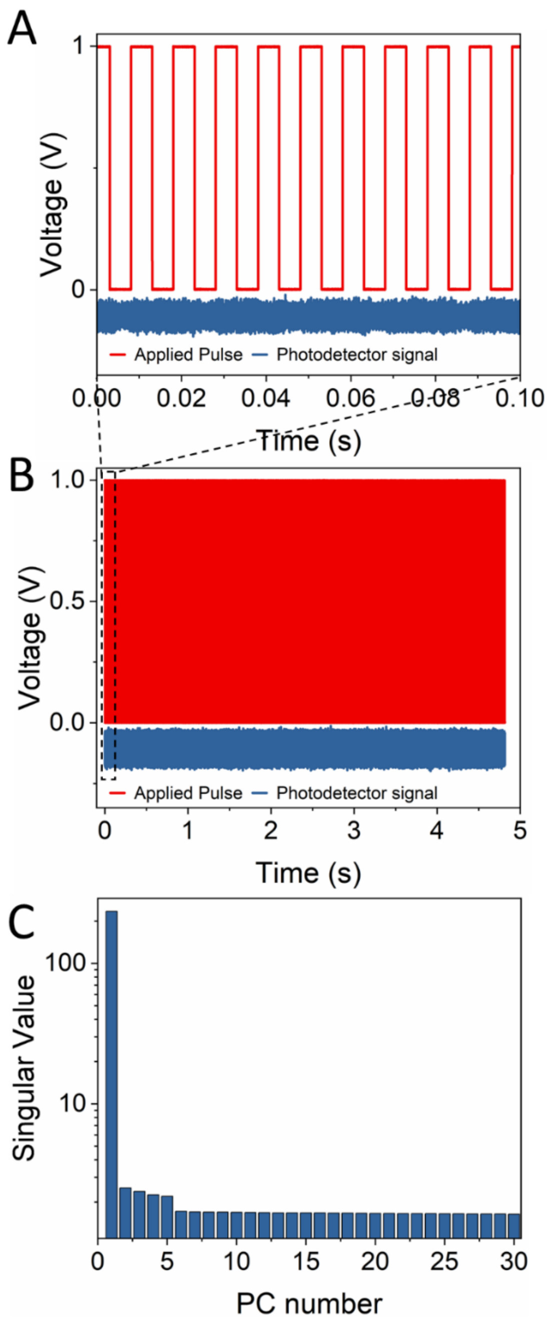

Fig. S2. Captured photodetector signal and corresponding scree plot for PCs. (A, B) The captured raw photodetector and applied pulses captured for 4.81 s, corresponding to 481 pulses of 10 ms pulse length. (C) Corresponding scree plot showing the five most significant PCs, beyond which the singular values of the components (referred to as noise components) lie on a straight line on the logarithmic plot.

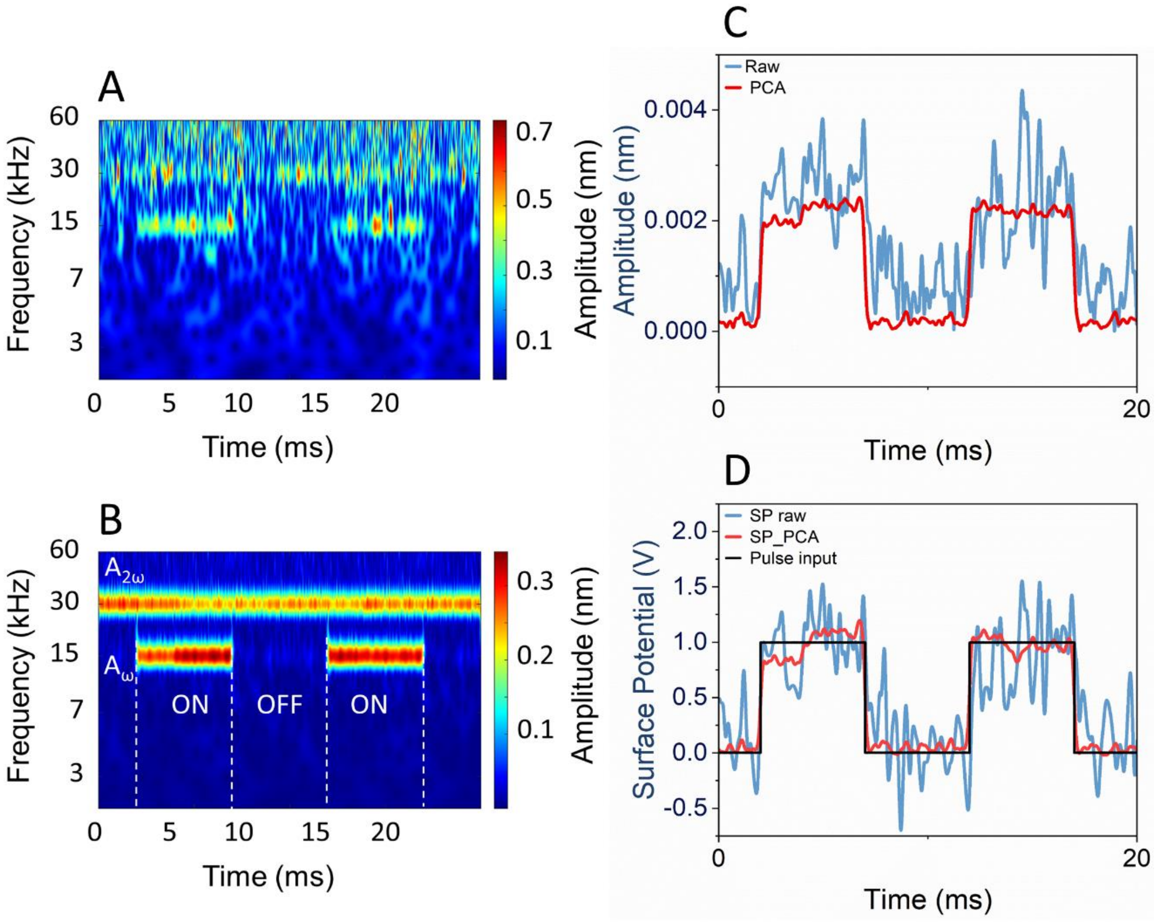

Fig. S3. PCA-led denoising of the raw photodetector signal. (A) CWT-derived amplitude scalograms of the raw photodetector signal for 10 ms pulse period, and (B) the corresponding PCA-led denoised CWT-derived scalogram. The temporal variation of the (C) extracted raw- and PCA-based denoised amplitude of the first harmonic, A_{ω}, (at 15 kHz) and (D) the calculated surface potential which highlights the reduction in the fluctuations and shows OL-WT-KPFM can track the applied pulse closely.

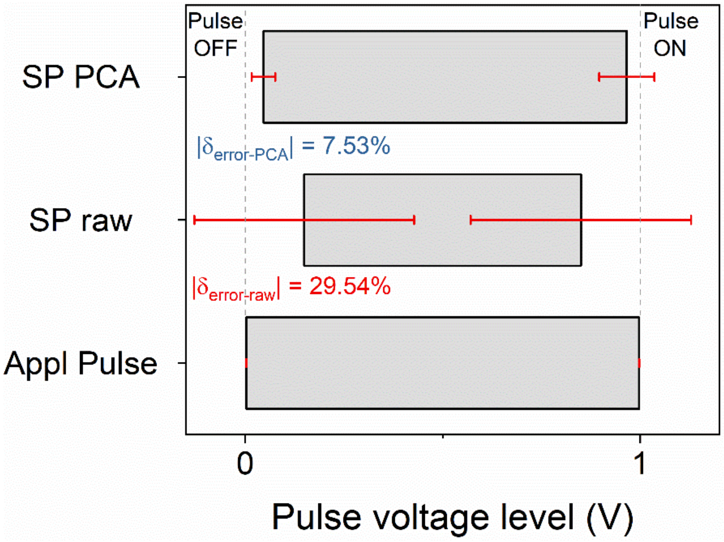

Fig. S4. Error quantification for raw and PCA-denoised signal. Extracted V_{CPD} values for the raw and PCA-based denoised signal shown in Fig. S3. The error is computed against the applied 1 V pulse, with the error bars denoting the standard deviation from 0 V (pulse OFF) and 1 V (pulse ON) levels, respectively.

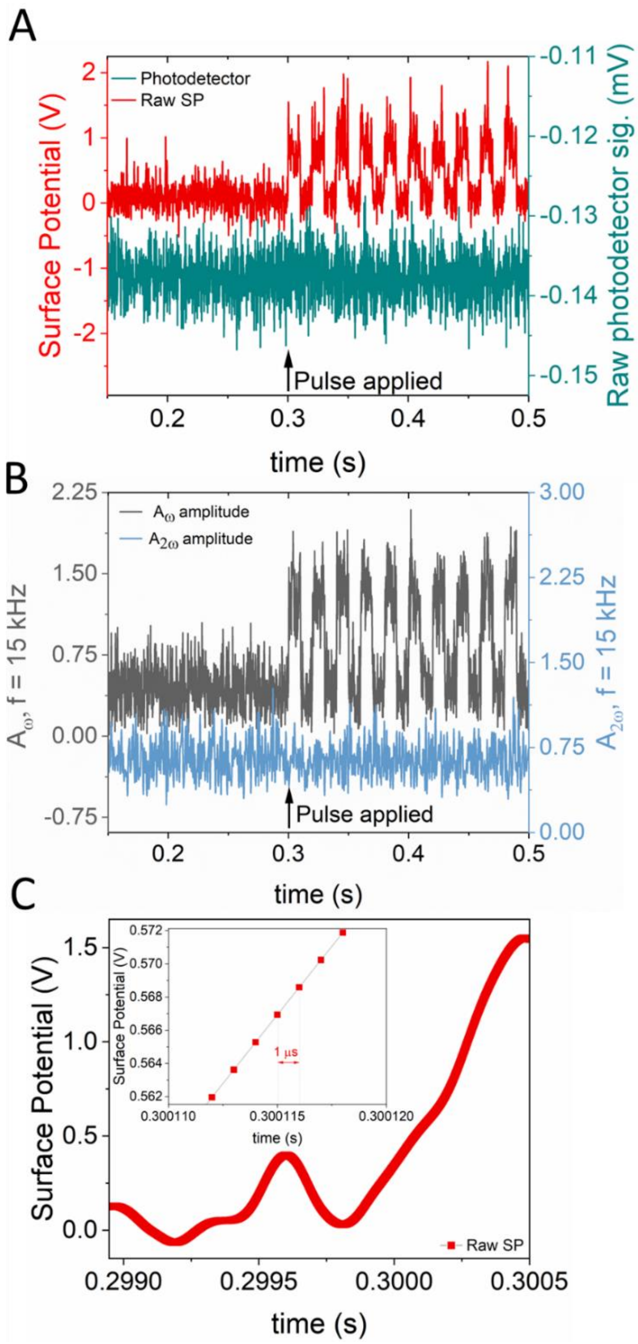

Fig. S5: Temporal resolution of OL-WT-KPFM. (A) The real-time photodetector signal acquired by the DAQ (bottom) with the corresponding raw surface potential values calculated *via* the OL-WT-KPFM method (prior to PCA denoising). (B) The calculations of the first A_ω and the second harmonic $A_{2\omega}$ at the AC electrical drive frequency. (C) 1 µs time resolution for the surface potential calculations.

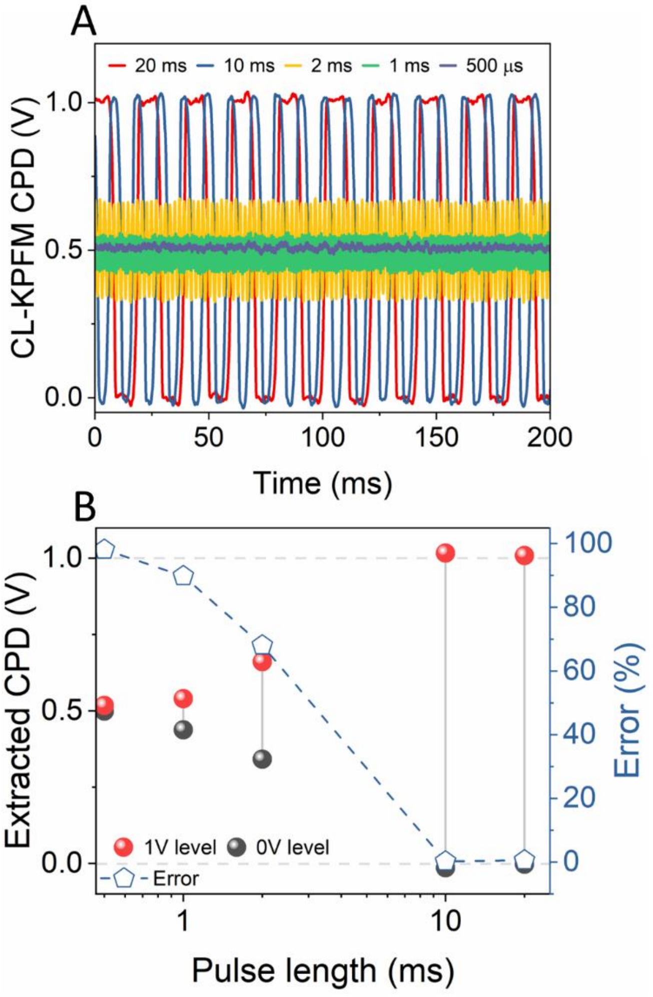

Fig. S6. CL-KPFM pulse data and error values. (A) Optimised CL-KPFM data for applied pulse periods of 20 ms – 500 μs. (B) The recovered SP from CL-KPFM shows significant deviations from the applied pulse (1V) as the pulse length approaches the feedback loop time constant (and cantilever bandwidth). The corresponding error in the extracted V_{CPD} jumps dramatically from ~1% for a 10 ms pulse length to ~70% for the 2 ms pulse length, respectively.

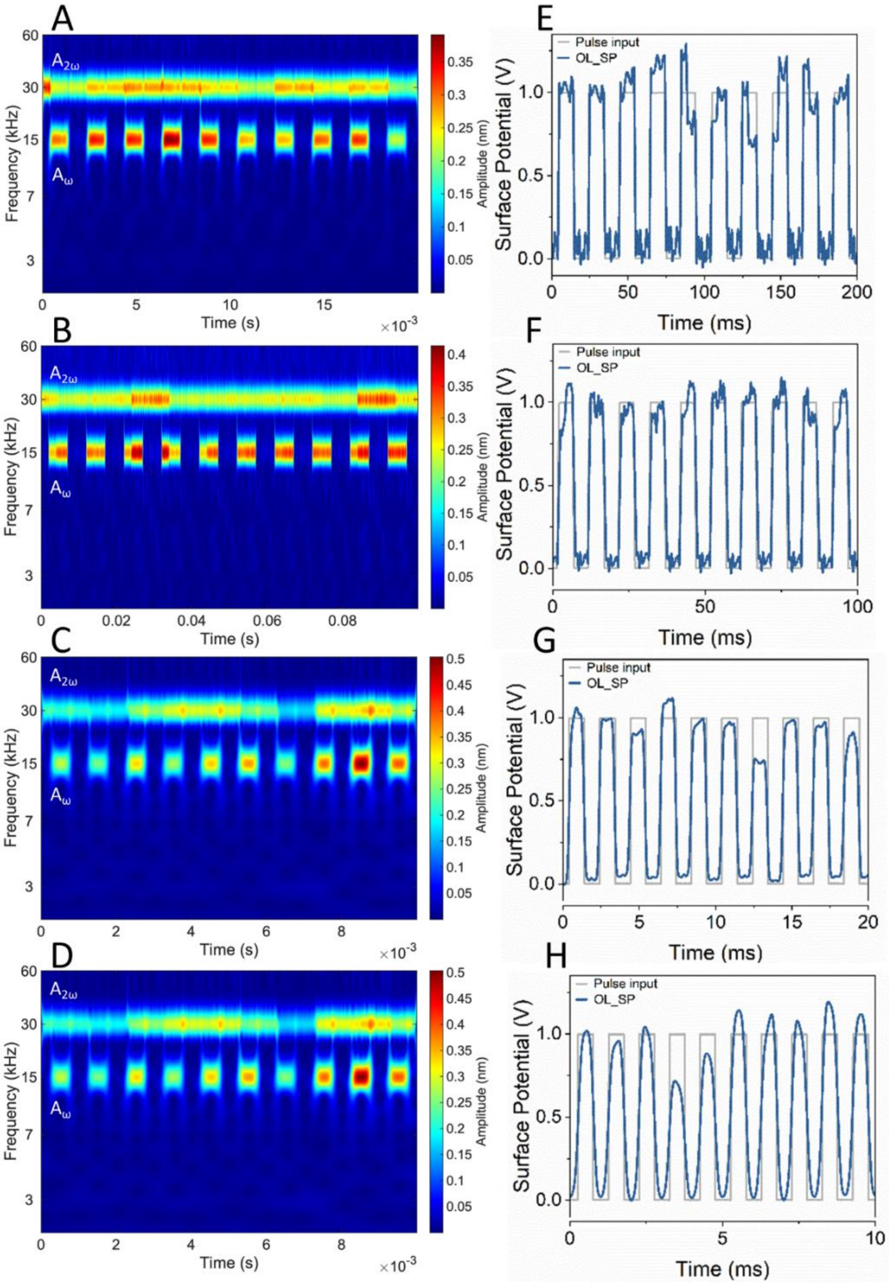

Fig. S7. CWT-derived amplitude scalograms and recovered SP values. Amplitude scalograms of the raw photodetector signal for the 0-1 V pulse signal of (A) 20 ms, (B) 10 ms, (C) 2 ms, and (D) 1 ms pulse period, respectively. (F-H) The corresponding comparison of recovered surface potential and the applied input pulse which highlights the advantage of the OL-WT-KPFM technique wherein the technique can detect and quantify the applied pulse signals even beyond the cantilever bandwidth, albeit with lower accuracy.

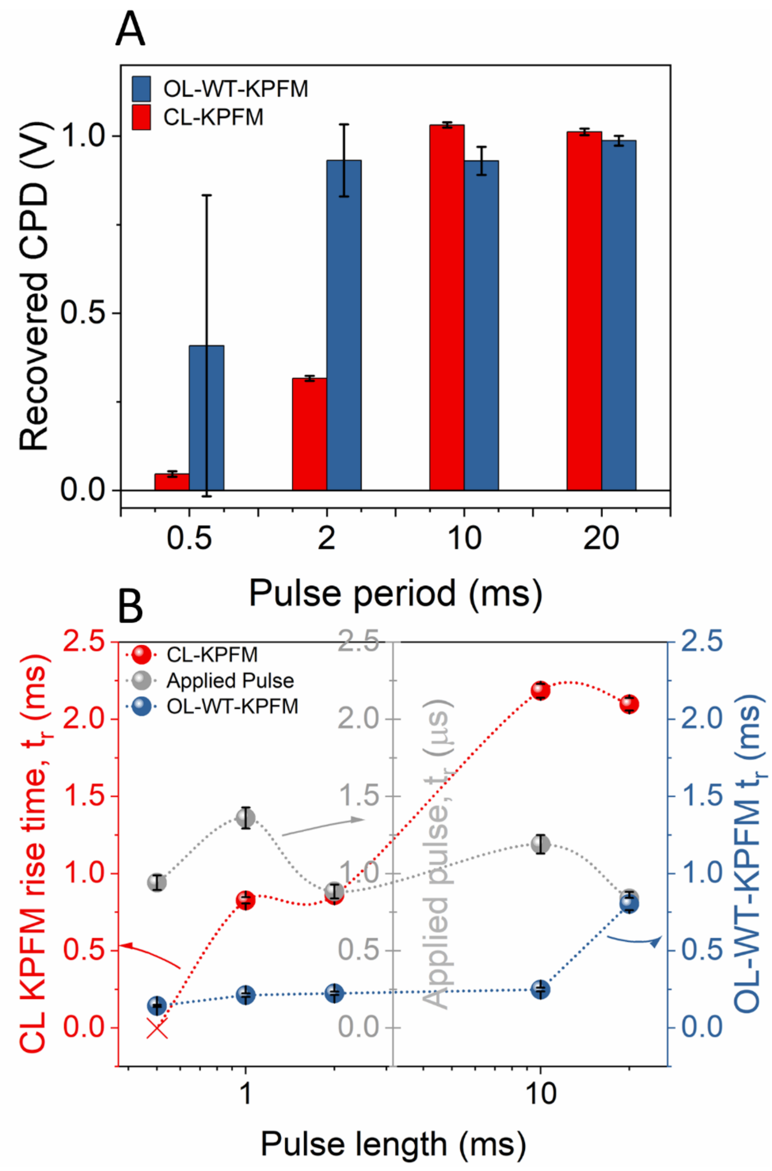

Fig. S8. Accuracy of CL-KPFM *vs.* OL-WT-KPFM methods. (A) Comparison of the recovered CPD values for the CL-KPFM and OL-WT-KPFM methods *vs.* pulse period, respectively. It can be clearly seen that while OL-WT-KPFM is able to extract the CPD with high accuracy beyond the limitation of the feedback loop time constant, it is ultimately constrained by the cantilever bandwidth. (B) Comparison of the measured rise times (t_r) for the applied pulses vs. the measured CL-KPFM and OL-WT-KPFM methods which clearly show a 3-order difference between the two.

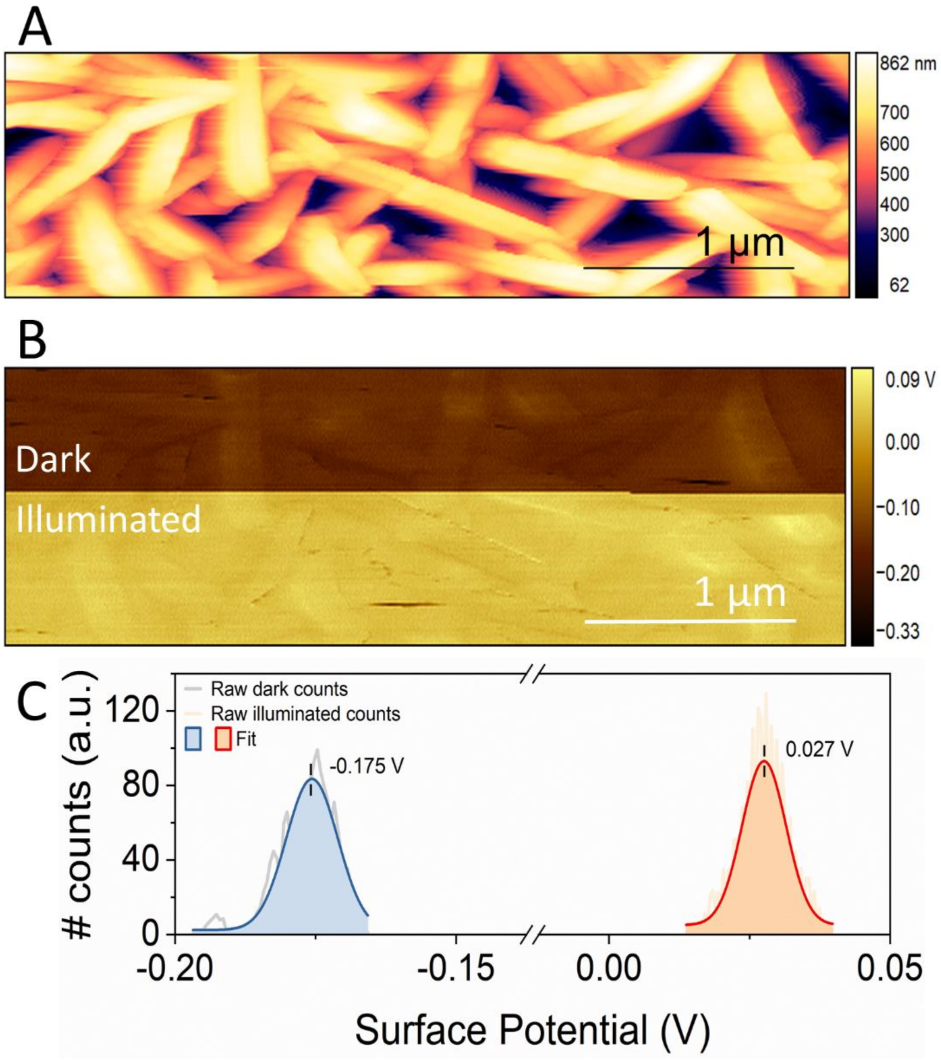

Fig. S9. CL-KPFM based imaging of BiOI (A) topography, and SP under (B) dark and illuminated conditions, respectively. The corresponding histogram (solid lines denote fitted curves) of the SP images is shown in (C) which shows the clear shift in the SP upon illumination.

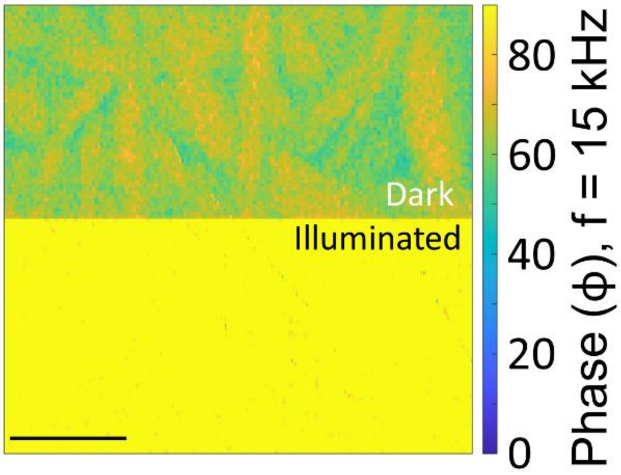

Fig. S10. OL-WT-KPFM extracted phase data. Phase data for the BiOI sample under dark and illuminated conditions. The horizontal scale bar represents 1 μm.

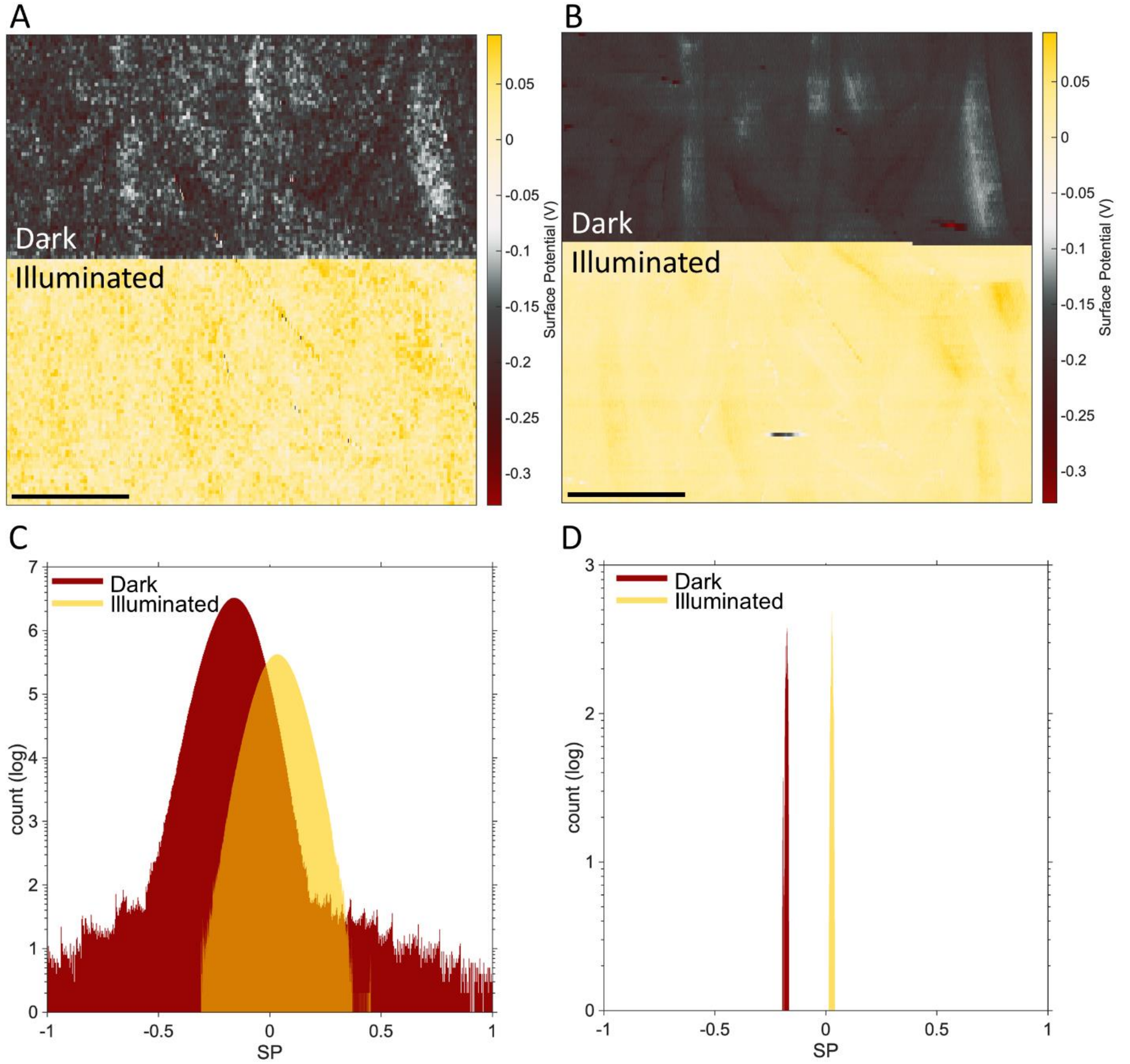

Fig. S11. Comparison of CL-KPFM and OL-WT-KPFM derived SP maps. The (A) OL-WT-KPFM derived SP map for the dark and illuminated conditions shows high consistency of the features with (B) the corresponding CL-KPFM image of BiOI. (C) A significantly wider SP histogram distribution shows the higher sensitivity of the OL-WT-KPFM technique as compared to the (D) CL-KPFM technique. The horizontal scale bar in (A, B) represents 1 μm.

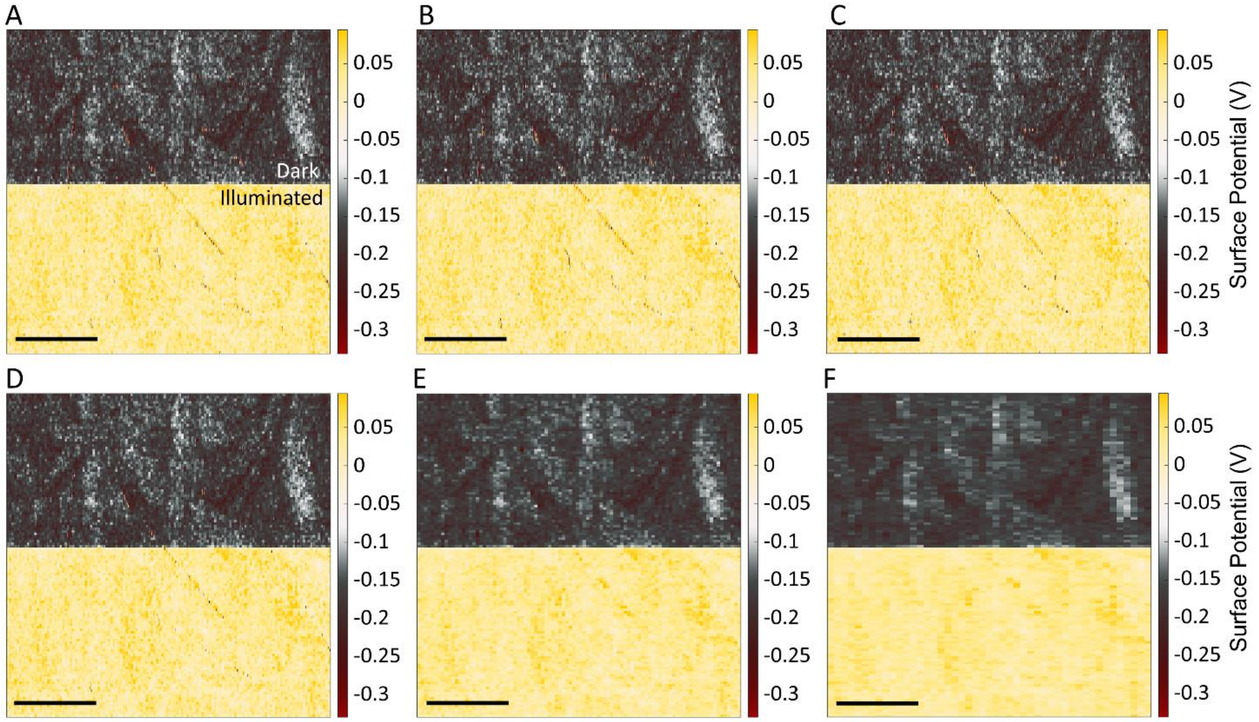

Fig. S12. Spatio-temporal evolution of the surface photovoltage of BiOI. Variation of surface potential after the switching ON of the illumination condition, at various time-averaged values of (A) 1 μs, (B) 10 μs, (C) 100 μs, (D) 1 ms, (E) 10 ms and (F) 20 ms, respectively. The horizontal scale bar represents 1 μm.

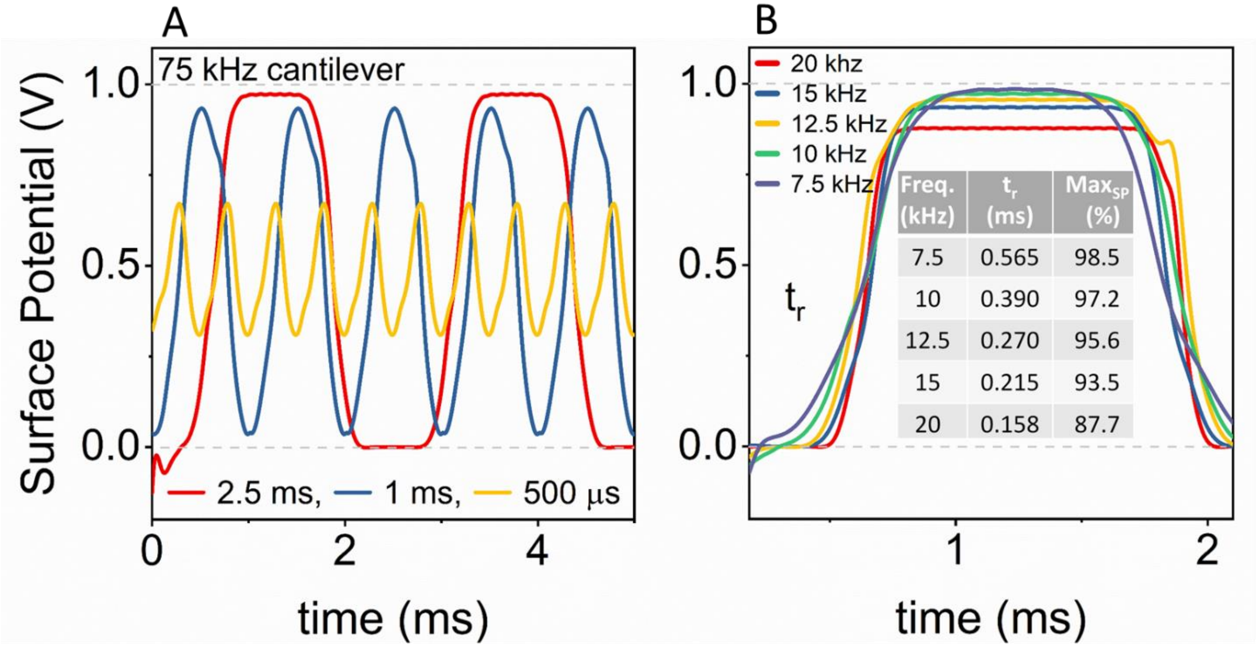

Fig. S13. Numerical simulation of the accuracy of the proposed OL-WT-KPFM method. (A) Simulation results of the calculated SP for the nominal 75 kHz cantilever (used in the experiments) for three different pulses with a square wave period of 2.5 ms, 1 ms, and 500 μs. (B) Reconstructed SP for the transient pulse with 1 ms time period considering different frequencies for the AC drive signal. The simulation parameters of the cantilever are $k = 2.59, Q = 250, C'_z = 1 \times 10^{-9}, V_{AC} = 1.5V, V_{CPD} = 1V$. The natural resonant frequencies are ω_0 = 75, 150, 300, 600 and 1200 kHz, respectively.

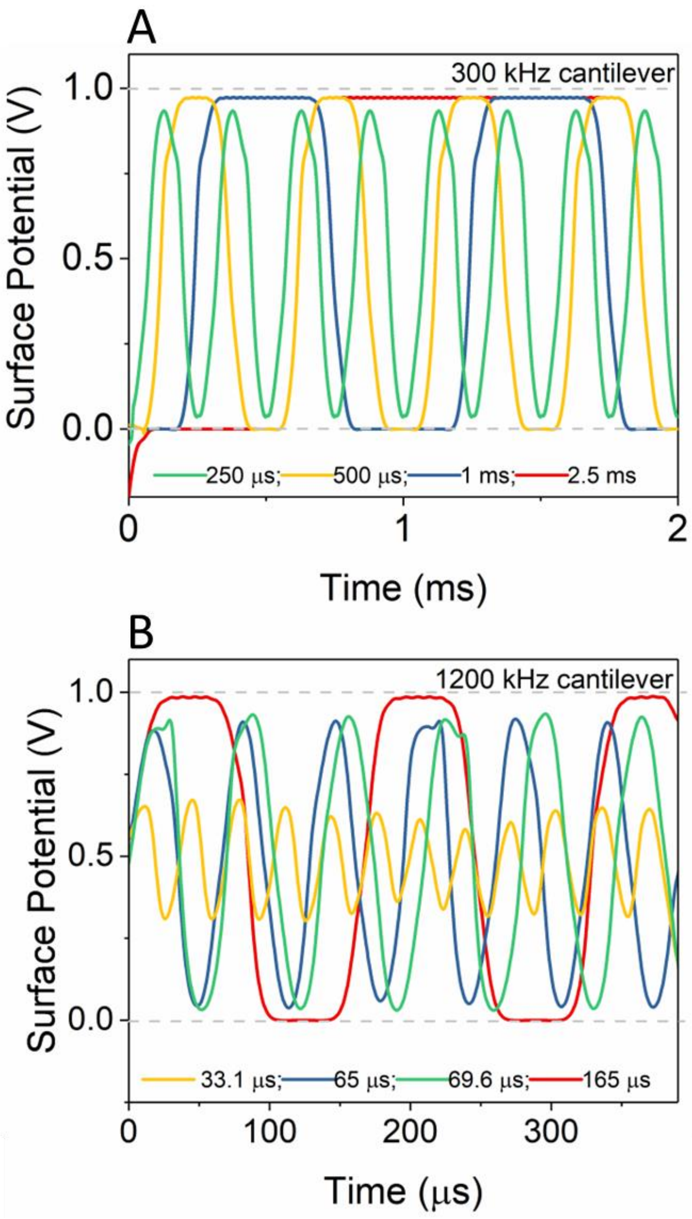

Fig. S14. Simulation of a 300 and 1200 kHz cantilever subjected to varying pulses. Calculated SP for (A) a 300 kHz cantilever for different pulses with periods of 2.5 ms, 1 ms, 500 μs and 250 μs, and (B) a 1200 kHz cantilever for pulses with a period of 33.1μs, 65 μs, 69.6 μs, and 165 μs, respectively.

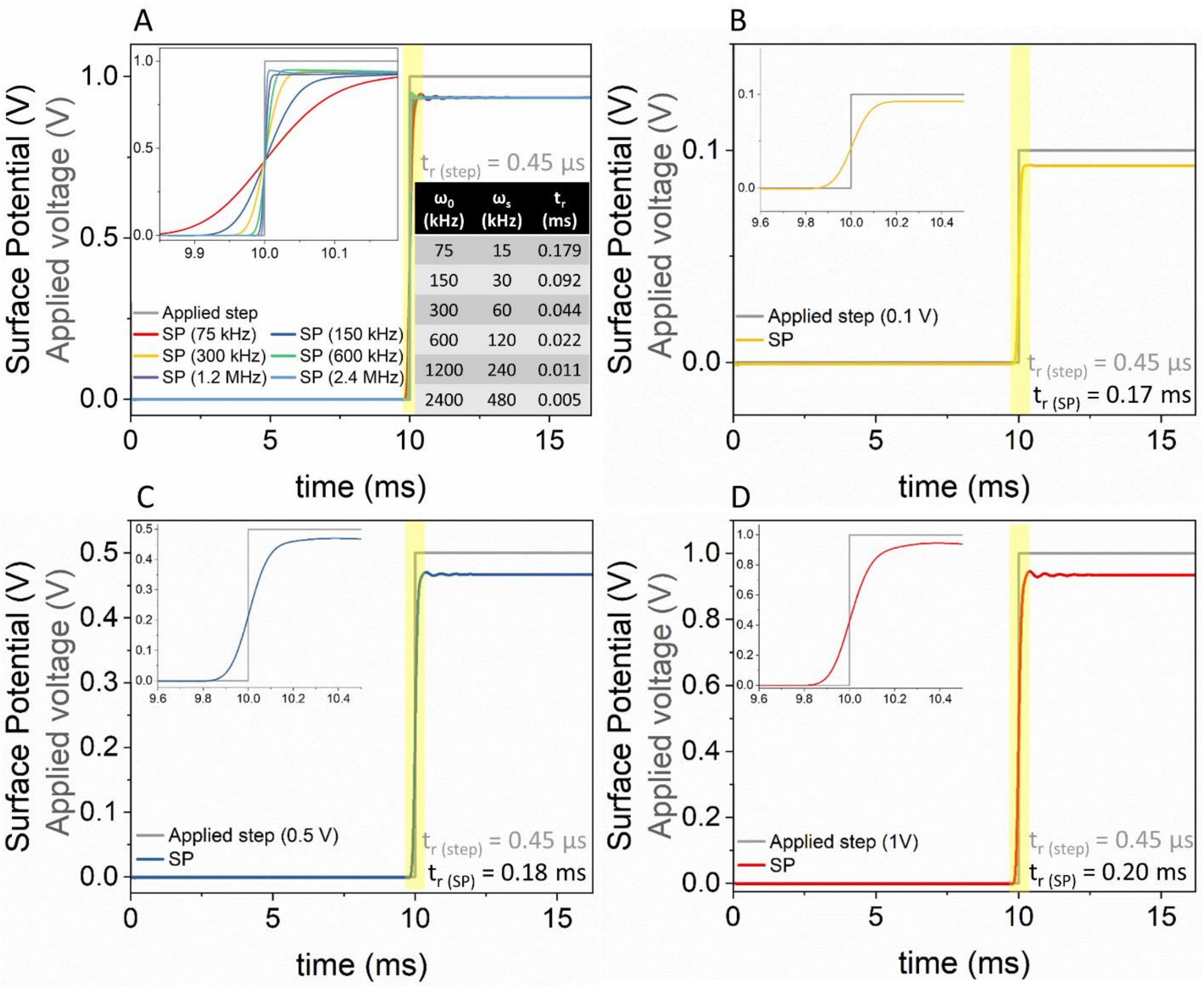

Fig. S15. Simulation results of the calculated SP and rise time (t_r). (A) Rise in SP in response an applied step input of 1V for cantilevers with different resonant frequencies, and (B-D) with varying amplitude of the step signal, respectively.

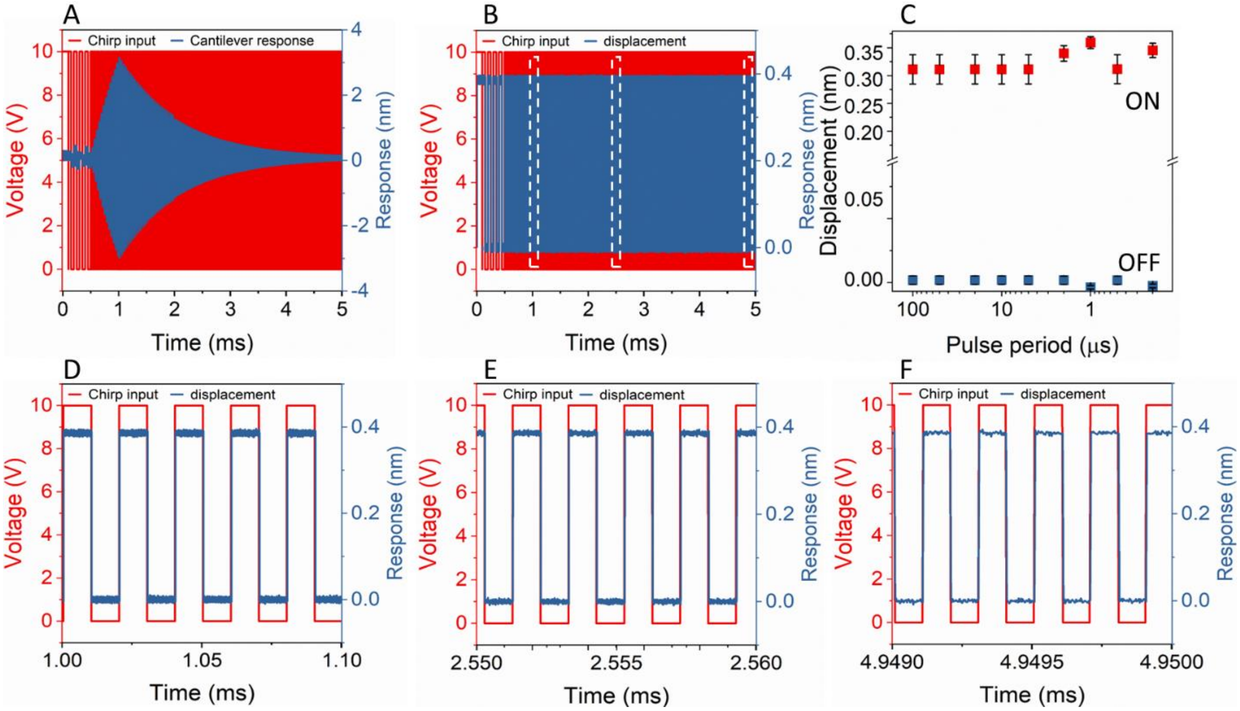

Fig. S16. Time resolution of the WT technique. (A) Reconstructed tip displacement (blue) to an applied chirp waveform (red) in which the pulse period was swept from ~0.1 ms to ~200 ns over a window of 5 ms (B) Recovered displacement with short time segments of the displacement (indicated on B) having (D) ~20 μs, (E) ~1 μs, and (F) ~200 ns pulse lengths are shown. (C) Plot showing the mean and standard deviation (within central 80% of the pulse width) for on (red) and off (blue) bias states.

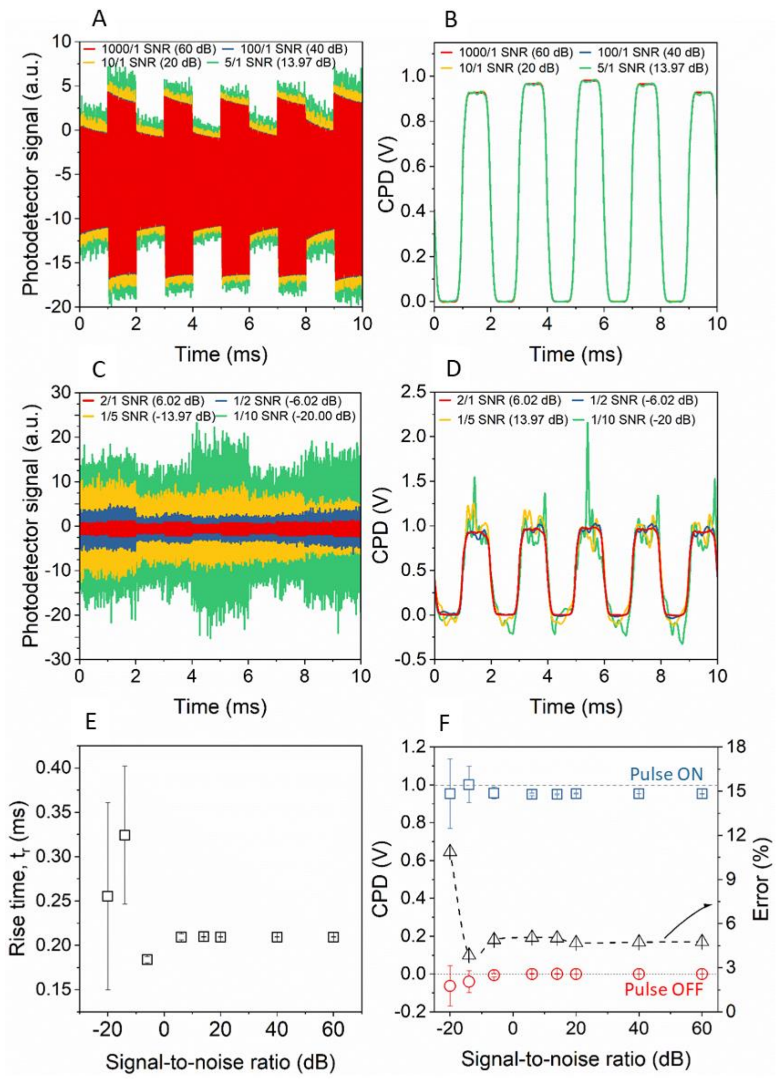

Fig. S17. Effect of noise on CPD extraction *via* WT. (A, C) Simulated signal with SNR levels ranging from 1000/1 SNR (60 dB) to 1/10 SNR (-20.00 dB). The corresponding (B, D) extracted V_{CPD} curves and (E) rise times, show the ability of the WT method to isolate temporal information from signals even with very high noise levels. (F) The extracted V_{CPD} values with the corresponding ON/OFF voltage levels and the variation of error wherein even for the highest SNR values (-20 dB), the error in the extracted CPD was ~10% only. For subfigure (F), the open circles represent the OFF state of the pulse while the open squares correspond to the ON state of the pulse and finally, the open triangles correspond to the computed error with respect to the applied 1 V pulse.

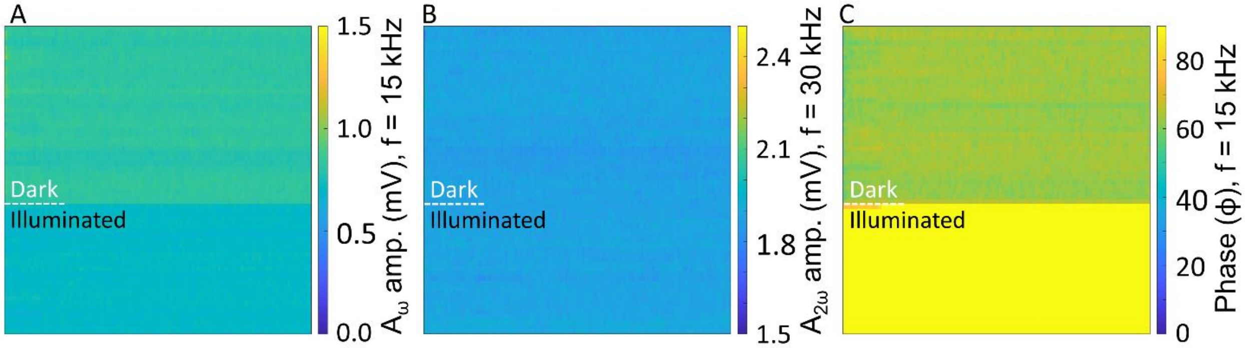

Fig. S18. Extraction of harmonics and phase from photodetector data stream. The CWT extracted (A) first harmonic (A_{ω}), (B) second harmonic ($A_{2\omega}$) and the corresponding (C) phase (φ_{ω}) from the single-pixel scan on BiOI. The horizontal dotted line shows when the illumination was switched ON. .

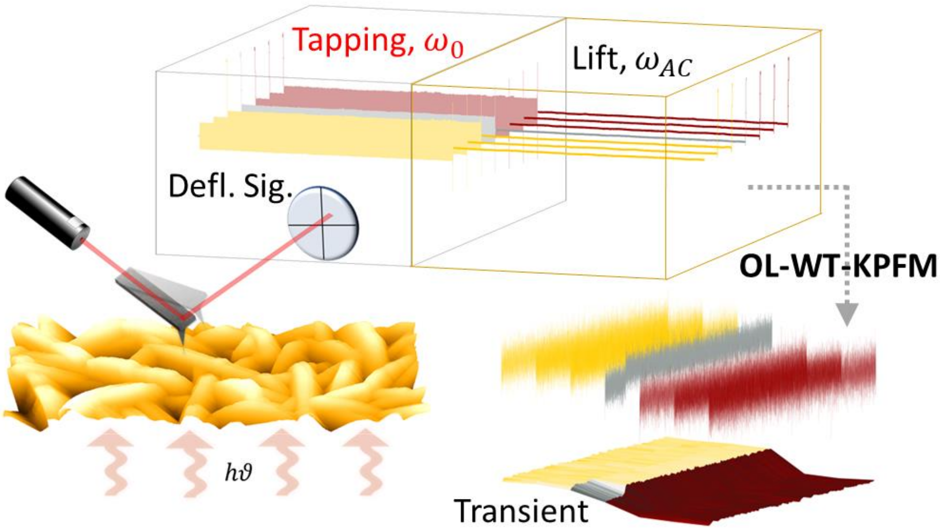

Fig. S19. Converting photodetector signal to image. The raw photodetector data captures both the tapping- and lift-mode signals in 1D. Initially the 1D signal is reshaped by considering the scanning rate to form the 2D image, while each tapping and lift section make up one line of the image. For extracting SP and transient (unavailable in CL-KPFM), the tapping portion of the signal is discarded while the lift portion of the signal is processed. Herein, each segment of the lift is one line of the image and by applying the OL-WT-KPFM methodology, the SP and transient behaviour is captured

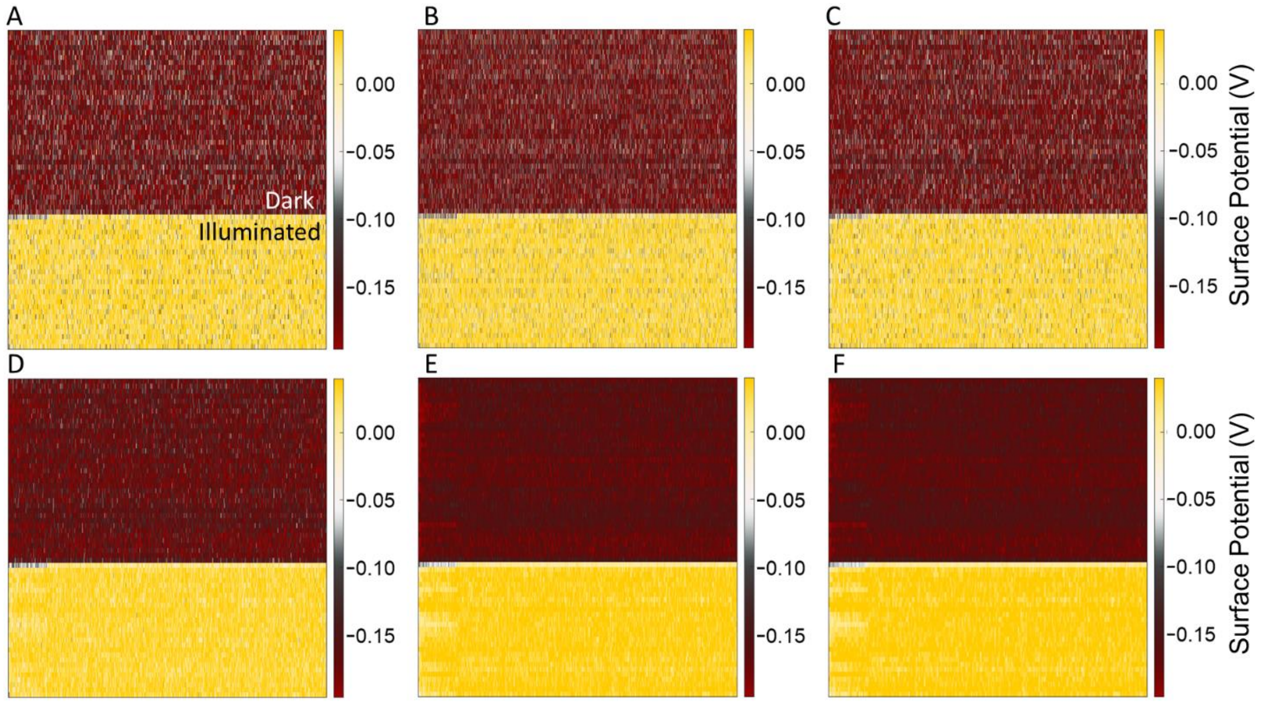

Fig. S20. CWT-derived SP maps for single pixel BiOI transient. Single pixel measurement of surface photovoltage induced change in surface potential of BiOI for switching on condition, at various time averaged values of (A) 1 μs, (B) 10 μs, (C 100 μs, (D) 1 ms, (E) 10 ms and (F) 20 ms, respectively.

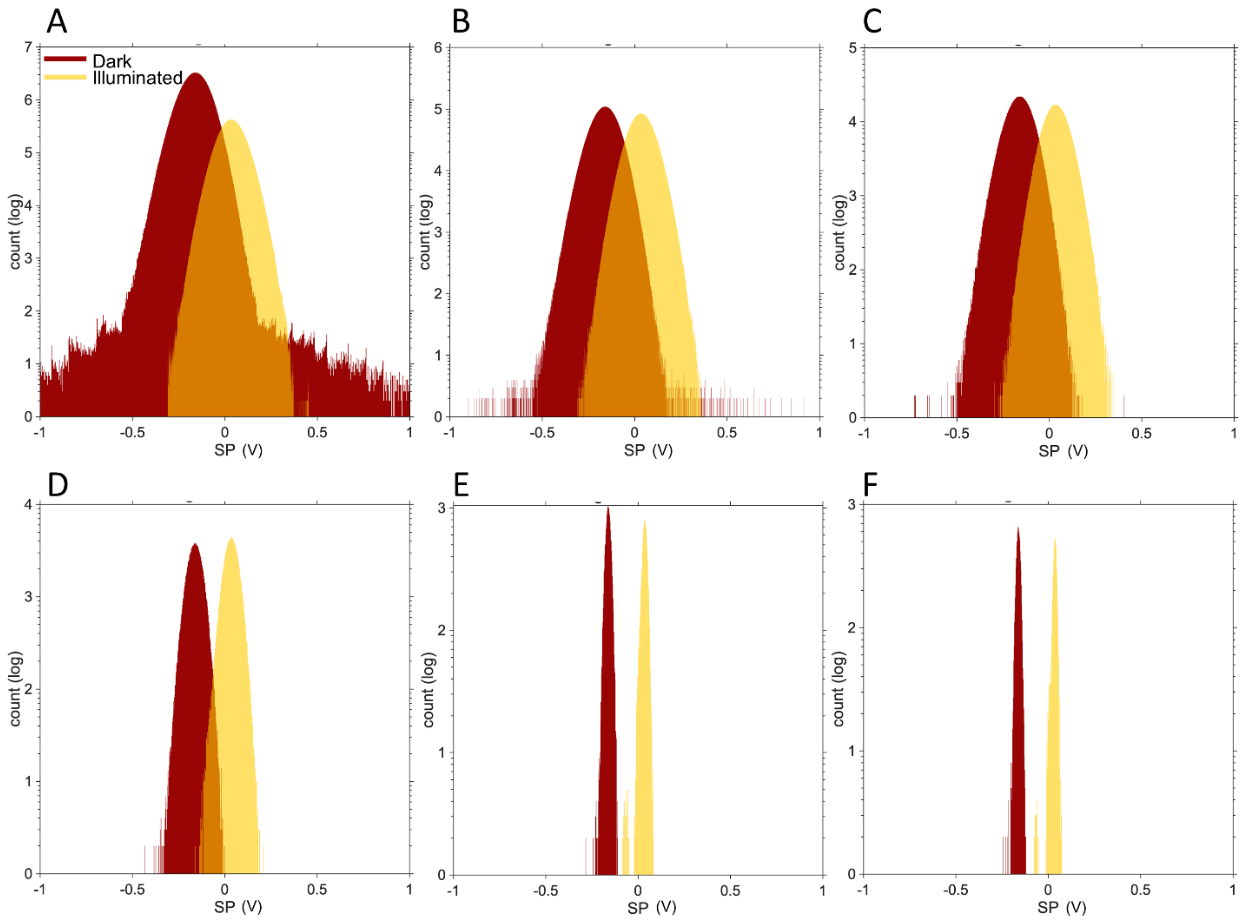

Fig. S21. Histograms of the CWT-derived SP amplitudes. The SP distribution for single-pixel BiOI OFF/ON transient at (A) 1 μs, (B) 10 μs, (C) 100 μs, (D) 1 ms, (E) 10 ms and (F) 20 ms, respectively.

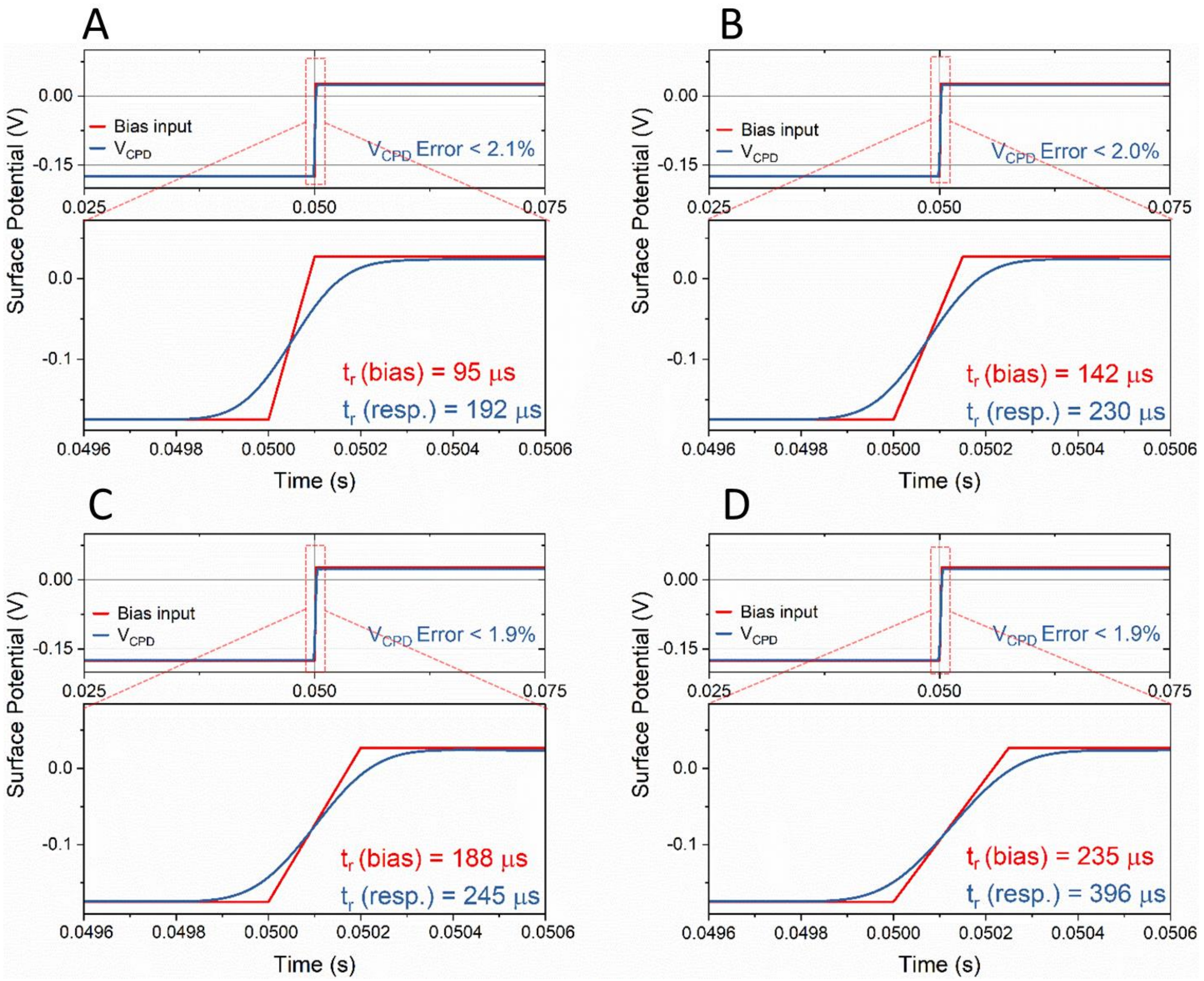

Fig. S22. Simulations for transient response time quantification at ω_{AC} = 25 kHz. The simulations for quantifying the transient response times and recovery of SP upon the surface photovoltage changes occurring in BiOI (modelled by a bias signal and ω_{AC} = 25 kHz) for (A) 100 µs, (B) 150 µs, (C) 200 µs and (D) 250 µs, respectively. Irrespective of the bias pulse rise time an absolute SP quantification error of less than 2% was observed.

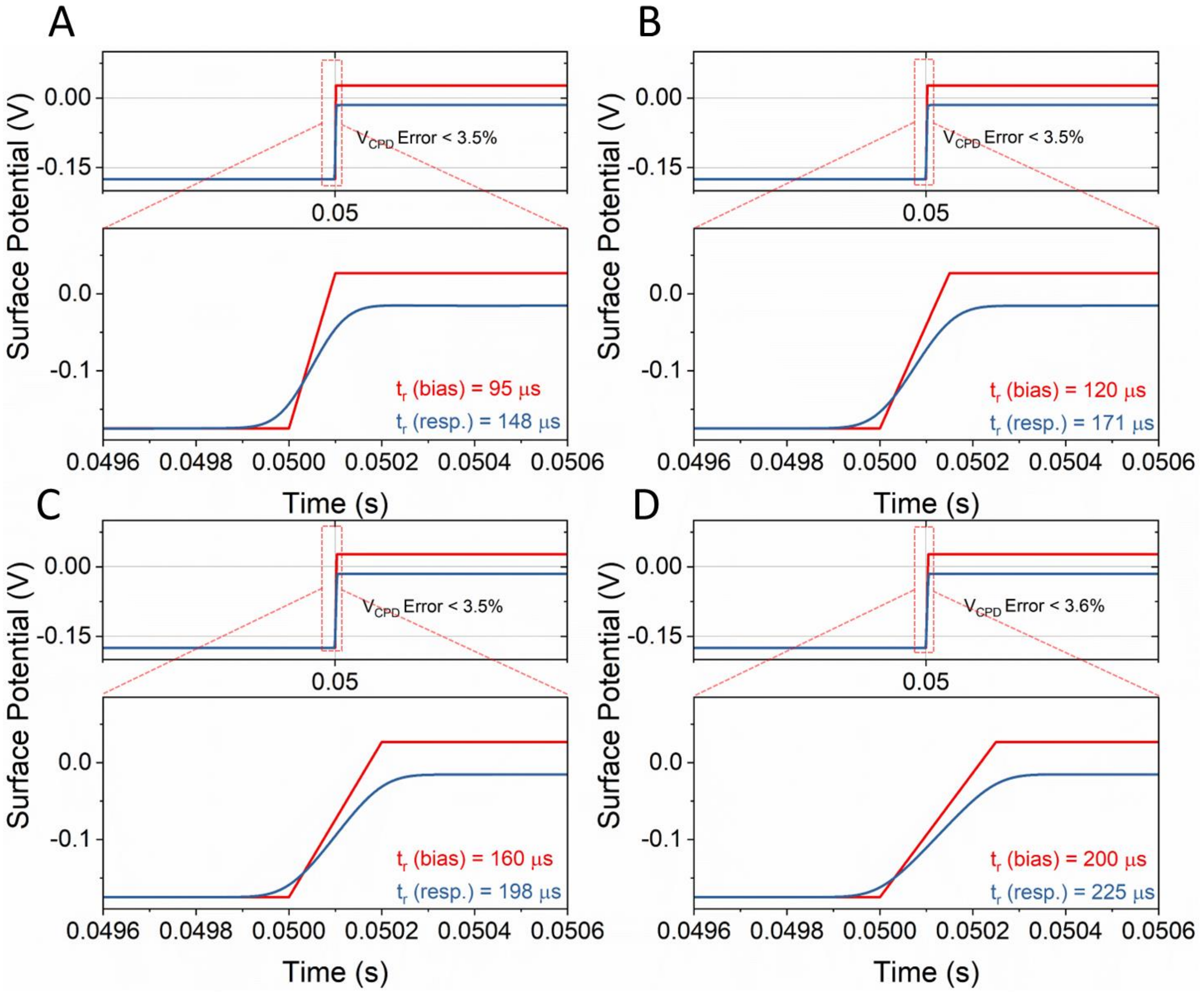

Fig. S23. Simulations for transient response time quantification at ω_{AC} = 10 kHz. The simulations for quantifying the transient response times and recovery of SP upon the surface photovoltage changes occurring in BiOI (modelled by a bias signal and ω_{AC} = 10 kHz) for (A) 100 μs, (B) 150 μs, (C) 200 μs and (D) 250 μs, respectively. Irrespective of the bias pulse rise time an absolute SP quantification error of less than 3.5% was observed.

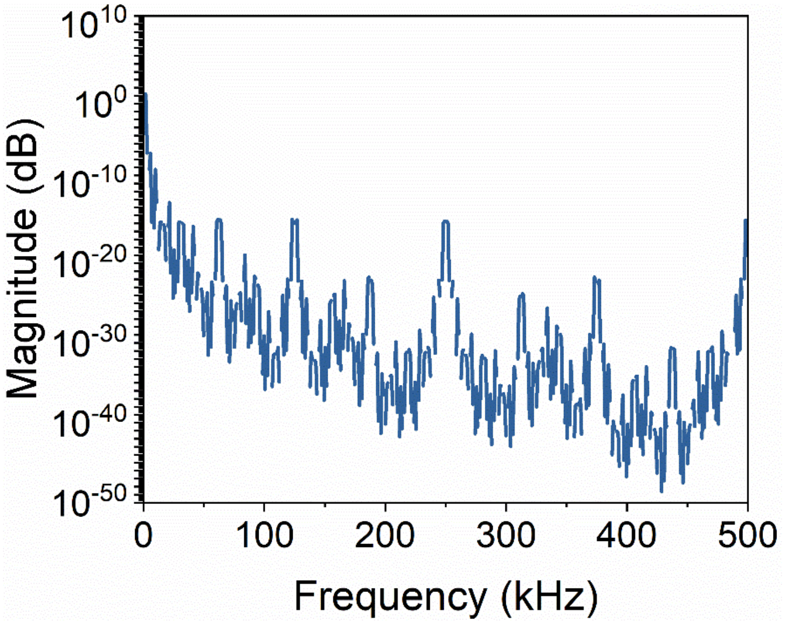

Fig. S24. Magnitude response of DWT. db45 9th level approximation.

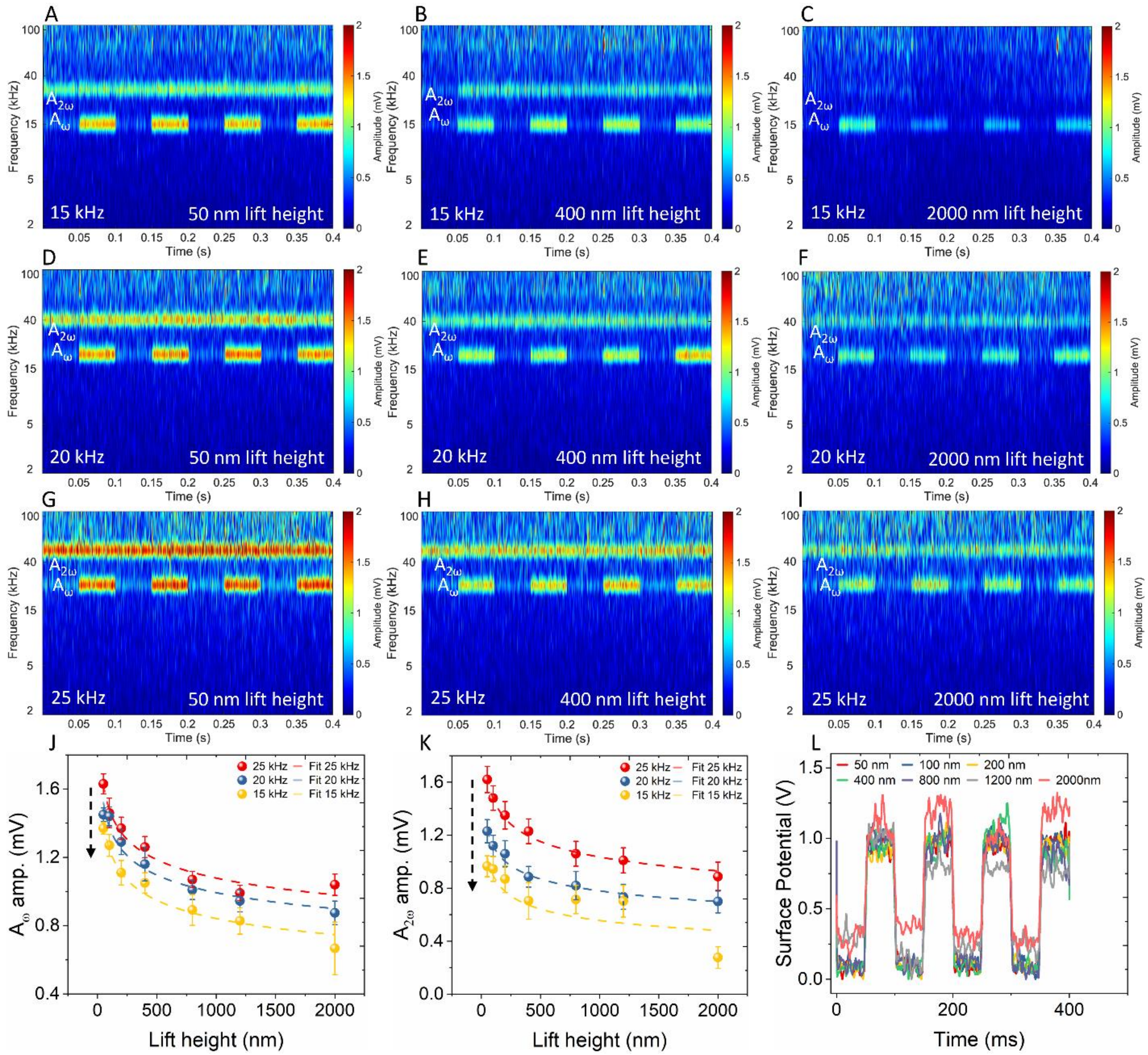

Fig. S25. Effect of lift height and choice of ω_{AC}. CWT-derived amplitude scalograms of the raw photodetector signal for the 50% duty cycle, 1 $V_{p\text{-}p}$ pulse signal applied on the Au pad. The scalograms show the temporal variation of the extracted A_{ω} and $A_{2\omega}$ channels for 15 kHz applied AC signal at (A) 50 nm, (B) 400 nm, (C) 2000 nm lift height, respectively. The variation of A_{ω} and $A_{2\omega}$ for 20 kHz AC signal at (D) 50 nm, (E) 400 nm, (F) 2000 nm lift height, respectively. The corresponding variations for the 25 kHz AC signal are shown in at (G) 50 nm, (H) 400 nm, (I) 2000 nm lift height, respectively. The absolute amplitudes of the (J) A_{ω} and (K) $A_{2\omega}$ harmonics

reduce as a function of applied AC signal and the lift height. (L) SP calculations for the case of 25 kHz applied AC signal as a function of lift height.

**SNR (dB)**	**60**	**40**	**20**	**13.98**	**6.02**	**-6.02**	**13.98**	**-20**
**Raw signal**	0.002	0.022	0.265	0.463	1.253	4.892	11.821	26.528
**Signal with PCA filtering**	0.001	0.017	0.122	0.341	0.714	2.691	7.366	20.807

Table S1. Relative error of the second harmonic amplitude (%)

References

(1) Kilpatrick, J. I.; Collins, L.; Weber, S. A. L.; Rodriguez, B. J. Quantitative Comparison of Closed-Loop and Dual Harmonic Kelvin Probe Force Microscopy Techniques. *Review of Scientific Instruments* **2018**, *89* (12). https://doi.org/10.1063/1.5025432.

(2) Collins, L.; Ahmadi, M.; Wu, T.; Hu, B.; Kalinin, S. V.; Jesse, S. Breaking the Time Barrier in Kelvin Probe Force Microscopy: Fast Free Force Reconstruction Using the G-Mode Platform. *ACS Nano* **2017**, *11* (9), 8717–8729. https://doi.org/10.1021/acsnano.7b02114.

(3) Zhang, C.; Han, R.; Zhang, A. R.; Voyles, P. M. Denoising Atomic Resolution 4D Scanning Transmission Electron Microscopy Data with Tensor Singular Value Decomposition. *Ultramicroscopy* **2020**, *219*, 113123. https://doi.org/10.1016/J.ULTRAMIC.2020.113123.

(4) Gu, X.; Li, G.; Zhou, M.; Lo, K. L. Wavelet Transform Based Approach to Harmonic Analysis. *Proceeding of the International Conference on Electrical Power Quality and Utilisation, EPQU* **2011**, 304. https://doi.org/10.1109/EPQU.2011.6128954.

(5) van Drongelen, W. Modeling Neural Systems. *Signal Processing for Neuroscientists* **2018**, 619–646. https://doi.org/10.1016/B978-0-12-810482-8.00029-1.

(6) Liscio, A.; Palermo, V.; Müllen, K.; Samorì, P. Tip - Sample Interactions in Kelvin Probe Force Microscopy: Quantitative Measurement of the Local Surface Potential. *Journal of Physical Chemistry C* **2008**, *112* (44), 17368–17377. https://doi.org/10.1021/JP806657K/ASSET/IMAGES/JP-2008-06657K_M011.GIF.

(7) Hackl, T.; Schitter, G.; Mesquida, P. AC Kelvin Probe Force Microscopy Enables Charge Mapping in Water. *ACS Nano* **2022**, *16* (11), 17982–17990.

https://doi.org/10.1021/ACSNANO.2C07121/ASSET/IMAGES/LARGE/NN2C07121_0006.JPEG.

(8) Söngen, H.; Rahe, P.; Neff, J. L.; Bechstein, R.; Ritala, J.; Foster, A. S.; Kühnle, A. The Weight Function for Charges - A Rigorous Theoretical Concept for Kelvin Probe Force Microscopy. *J Appl Phys* **2016**, *119* (2), 25304. https://doi.org/10.1063/1.4939619/143547.

(9) Ma, Z. M.; Mu, J. L.; Tang, J.; Xue, H.; Zhang, H.; Xue, C. Y.; Liu, J.; Li, Y. J. Potential Sensitivities in Frequency Modulation and Heterodyne Amplitude Modulation Kelvin Probe Force Microscopes. *Nanoscale Res Lett* **2013**, *8* (1), 1–6. https://doi.org/10.1186/1556-276X-8-532/FIGURES/4.
